# A 3-Component Mixture of Rayleigh Distributions: Properties and Estimation in Bayesian Framework

**DOI:** 10.1371/journal.pone.0126183

**Published:** 2015-05-20

**Authors:** Muhammad Aslam, Muhammad Tahir, Zawar Hussain, Bander Al-Zahrani

**Affiliations:** 1 A Department of Basic Sciences, Riphah International University, Islamabad, 44000, Pakistan; 2 Department of Statistics, Quaid-i-Azam University, 45320, Islamabad, 44000, Pakistan; 3 Department of Statistics, King Abdulaziz University, 21589, Jeddah, 80203, Saudi Arabia; Fondazione Edmund Mach, Research and Innovation Centre, ITALY

## Abstract

To study lifetimes of certain engineering processes, a lifetime model which can accommodate the nature of such processes is desired. The mixture models of underlying lifetime distributions are intuitively more appropriate and appealing to model the heterogeneous nature of process as compared to simple models. This paper is about studying a 3-component mixture of the Rayleigh distributionsin Bayesian perspective. The censored sampling environment is considered due to its popularity in reliability theory and survival analysis. The expressions for the Bayes estimators and their posterior risks are derived under different scenarios. In case the case that no or little prior information is available, elicitation of hyperparameters is given. To examine, numerically, the performance of the Bayes estimators using non-informative and informative priors under different loss functions, we have simulated their statistical properties for different sample sizes and test termination times. In addition, to highlight the practical significance, an illustrative example based on a real-life engineering data is also given.

## Introduction

The Rayleigh distribution has many real life applications in testing lifetime of an object whose lifetime depends upon its age. The Rayleigh distribution is often used in different fields of physics to model processes such as wave heights (Rattanapitikon [1] and Van Vledder et al. [2]), sound and light radiation (Siddiqui [3]), radio signals and wind power (Ahmed and Mahammed [4]), ultrasound image modeling (Chivers [5] and Burekhardt [6]) etc. It is also used to model lifetime in hours of tubes, resistors, networks, crystals, knobs, transformers, relays and capacitors in aircraft radar sets. The Rayleigh distribution is used to study the wind speeds over a year at wind turbine sites and the daily average wind speed. In all of above mentioned applications, it is not uncommon to assume that life of particular equipment does depend upon its age. On the other hand, this distribution has got valuable attention in the field of reliability theory and survival analysis, probability theory and operations research. Thus, to model the age dependent lifetimes of devices/ equipments, the Rayleigh distribution may be a suitable candidate distribution.

When the data are given only from overall mixture distributions then modeling these data as a mixture of some component distributions is known as direct application of the mixture models. Li [7] and Li and Sedransk [8, 9] discussed different features of two types of mixture models. If the component distributions of a mixture belong to same family, their mixture is known as a type-I mixture model. Otherwise, it is named as a type-II mixture model.Mixture models have been successfully applied in many areas such as engineering, physical sciences, chemical sciences, biological sciences, etc. To understand the need of using mixture models, imagine a practical situation of modeling lifetimes of certain electrical elements where the population of lifetimes may be divided into a number of components depending upon the possible reasons of failure. Several authors have used mixture modeling in different practical problems. For example, Harris [10] fitted mixture distributions to model the crime and justice data, Kanji [11] described wind shear data using mixture distributions, Jones and McLachlan [12] applied the mixture of normal and Laplace distributions to wind shear data.

Most of the researchers worked on the classical and the Bayesian analysis of 2-component mixture models. McCullagh [13] derived some conditions under which quadratic and polynomial Exponential models can be generated as mixtures of Exponential models. Sinha [14] used the Bayesian counterpart of the maximum likelihood estimates of the 2-component mixture model considered by Mendenhall and Hader [15]. Hebert and Scariano [16] compared the location estimators for Exponential mixtures under Pitman’s measure of closeness. Sultan et al. [17] investigated the properties of the 2-component mixture of inverse Weibull distributions. Saleem and Aslam [18] discussed the use of the informative and the non-informative priors for Bayesian analysis of the 2-component mixture of Rayleigh distributions. Also, Saleem et al.[19] presented the Bayesian analysis of the 2-component mixture of the Power distributions using the complete and censored sample. Kazmi et al. [20] described the Bayesian analysis for the 2-component mixture of Maxwell distributions.

In daily life, many types of data including simple data, grouped data, truncated data, censored data and progressively censored data are encountered. Censoring is an important and valuable aspect of the lifetime data. Censoring is a form of primary quality and missing life time data problems. A valuable account of censoring is given in Romeu [21], Gijbels [22] and Kalbfleisch and Prentice [23].

Motivated by above mentioned applications of mixture of Rayleigh distributions, we plan to have Bayesian analysis of a 3-component mixture of Rayleigh distributions with unknown mixing proportions. The parameters of component distributions are assumed to be unknown. Four different priors and three different loss functions are used for Bayesian analysis. In addition, we assume an ordinary type-I right censored sampling scheme.

The rest of the paper is organized as follows: The 3-component mixture of Rayleigh distributions is defined in Section 2. The expressions for posterior distributions using the non-informative and the informative priors are derived in Section 3. The elicitation ofhyperparameters, if unknown, is given in Section 4. In Section 5, the Bayes estimators and posterior risks using the uniform, the Jeffreys’, the inverted chi-square and the square root inverted gamma priors under squared error loss function (SELF), precautionary loss function (PLF) and DeGroot loss function (DLF) are presented. The limiting expressions of the Bayes estimators and their posterior risksare derived in Section 6. The simulation study and the real data application arepresented in Sections7 and 8, respectively.Finally, the conclusion of this study is given in Section 9.

## 3-Component mixture of the Rayleigh distributions

The probability density function (p.d.f.) and the cumulative distribution function (c.d.f.) of the Rayleigh distribution for a random variable *Y* are given by:

$$f_{m}\left(y;\;\lambda_{m}\right)=\frac{y}{\lambda_{m}^{2}}\exp\left(-\frac{y^{2}}{2\lambda_{m}^{2}}\right),\;y\geq 0,\;\lambda_{m}>0,\;m=1,\;2,\;3.$$ (1)

$$F_{m}\left(y\right)=1-\exp\left(-\frac{y^{2}}{2\lambda_{m}^{2}}\right),\;m=1,\;2,\;3,$$ (2)

where *λ*_*m*_ is the parameter ofthe Rayleigh distribution.

A finite 3-component mixture model with the unknown mixing proportions *p*_1_ and *p*_2_ is defined as:

$$f\left(y\right)=p_{1}f_{1}\left(y\right)+p_{2}f_{2}\left(y\right)+\left(1-p_{1}-p_{2}\right)f_{3}\left(y\right),\;p_{1},\;p_{2}\geq 0,\;p_{1}+p_{2}\leq 1$$ (3)

$$f\left(y;\;\lambda_{1},\;\lambda_{2},\;\lambda_{3},\;p_{1},\;p_{2}\right)=p_{1}\frac{y}{\lambda_{1}^{2}}\exp\left(-\frac{y^{2}}{2\lambda_{1}^{2}}\right)+p_{2}\frac{y}{\lambda_{2}^{2}}\exp\left(-\frac{y^{2}}{2\lambda_{2}^{2}}\right)+\left(1-p_{1}-p_{2}\right)\frac{y}{\lambda_{3}^{2}}\exp\left(-\frac{y^{2}}{2\lambda_{3}^{2}}\right).$$ (4)

**Fig 1:**
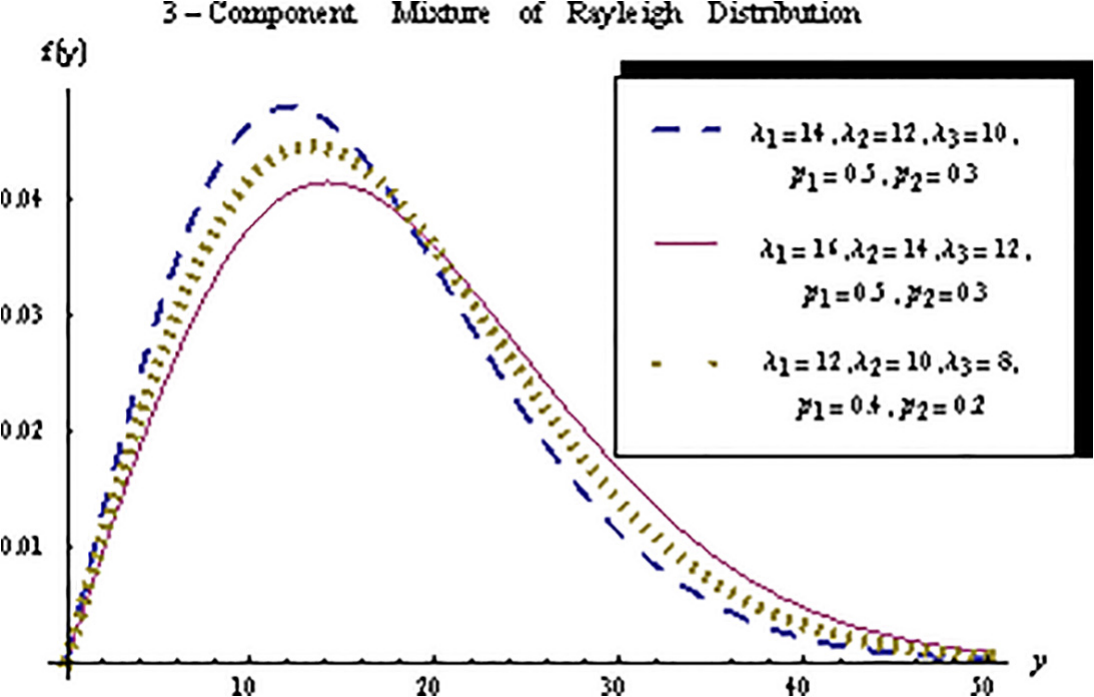
Graphs of 3-component mixture of the Rayleigh distributions for different values of parameters.

For different values of component and mixing proportion parameters, the behavior of a 3-component mixture of the Rayleigh distributions is depicted in the Fig 1.

The cumulative distribution function of 3-component mixture of the Rayleigh distributions is given by:

$$F\left(y\right)=p_{1}F_{1}\left(y\right)+p_{2}F_{2}\left(y\right)+\left(1-p_{1}-p_{2}\right)F_{3}\left(y\right)$$ (5)

$$F\left(y\right)=1-p_{1}\exp\left(-\frac{y^{2}}{2\lambda_{1}^{2}}\right)-p_{2}\exp\left(-\frac{y^{2}}{2\lambda_{2}^{2}}\right)-\left(1-p_{1}-p_{2}\right)\exp\left(-\frac{y^{2}}{2\lambda_{3}^{2}}\right).$$ (6)

## The posterior distribution using the non-informative and the informative priors

In this section, likelihood and posterior distributions of parameters given data, say **y**, are derived using the non-informative (uniform and Jeffreys’) and the informative (inverted chi-square and square root inverted gamma) priors.

### 3.1 The likelihood function

Suppose *n* units from the 3-component mixture of Rayleigh distributionsare used in a life testing experiment with fixed test termination time *t*. Let the experiment be performed and it is observed that *r* out of *n* units failed until fixed test termination time *t* and the remaining *n* − *r* units are still working. It is to be noted that out of *r* failures, *r*_1_, *r*_2_ and *r*_3_ failures can be categorized as belong to subpopulation-I, subpopulation-II and subpopulation-III, respectively, depending upon the reason of failure. So, the number of uncensored observations is *r* = *r*_1_+*r*_2_+*r*_3_. The remaining *n* – *r* observations are the censored observations. Now we define *y*_*lk*_, 0 < *y*_*lk*_ ≤ *t*, be the failure time of the *k* ^th^ unit belonging to the *l* ^th^ subpopulation, where *l* = 1, 2, 3 and *k* = 1, 2,⋯, *r*_*l*_. For a 3-component mixture model, the likelihood functioncan be written as:

$$L\left(\phi |y\right)\propto \left\{\prod_{k=1}^{r_{1}}p_{1}f_{1}\left(y_{1k}\right)\right\}\left\{\prod_{k=1}^{r_{2}}p_{2}f_{2}\left(y_{2k}\right)\right\}\left\{\prod_{k=1}^{r_{3}}\left(1-p_{1}-p_{2}\right)f_{3}\left(y_{3k}\right)\right\}{\left\{1-F\left(t\right)\right\}}^{n-r}$$ (7)

After simplification (see S1 File), the likelihood function of 3-component mixture of Rayleigh distributions is given by:

$$\begin{array}{l} L\left(\phi |y\right)\propto \lambda_{1}^{-2r_{1}}\lambda_{2}^{-2r_{2}}\lambda_{3}^{-2r_{3}}[\sum_{i=0}^{n-r}\sum_{j=0}^{i}\left(\begin{array}{l} n-r\\ \;i \end{array}\right)\left(\begin{array}{l} i\\ \text{j} \end{array}\right)\exp\left\{-\frac{1}{\lambda_{1}^{2}}\left(\left(n-r-i\right)\frac{t^{2}}{2}+\frac{1}{2}\sum_{k=1}^{r_{1}}y_{1k}^{2}\right)\right\}\exp\left\{-\frac{1}{\lambda_{2}^{2}}\left(\left(i-j\right)\frac{t^{2}}{2}+\frac{1}{2}\sum_{k=1}^{r_{2}}y_{2k}^{2}\right)\right\}\\ \exp\left\{-\frac{1}{\lambda_{3}^{2}}\left(j\frac{t^{2}}{2}+\frac{1}{2}\sum_{k=1}^{r_{3}}y_{3k}^{2}\right)\right\}p_{1}^{n-r-i+r_{1}}p_{2}^{i-j+r_{2}}{\left(1-p_{1}-p_{2}\right)}^{j+r_{3}}], \end{array}$$ (8)

where $y=\left(y_{11},\;y_{12},\ldots ,\;y_{1r_{1}},\;y_{21},\;y_{22},\ldots ,\;y_{2r_{2}},\;y_{31},\;y_{32},\ldots ,\;y_{3r_{3}}\right)$ are the observed failure times for the uncensored observations and **ϕ** = (*λ*_1_, *λ*_2_, *λ*_3_, *p*_1_, *p*_2_).

### 3.2 The posterior distribution usingthe uniform prior

The most common non-informative priors are the uniform prior (UP) and the Jeffreys’ prior (JP). Bayes [24], de Laplace [25] and Geisser [26] proposed that one may take the UP for the unknown parameters of interest.We assume the improper UP (which is proportional to a constant) for the component parameters *λ*_1_, *λ*_2_ and *λ*_3_, i.e., *λ*_1_ ∼ *Uniform*(0,∞), *λ*_2_ ∼ *Uniform*(0,∞) and *λ*_3_ ∼ *Uniform*(0,∞). The UP over the interval (0,1) is assumed for the proportion parameters *p*_1_ and *p*_2_, i.e., *p*_1_ ∼ *Uniform*(0,1) and *p*_2_ ∼ *Uniform*(0,1). Assuming the independence of parameters, the joint prior distribution of parameters *λ*_1_, *λ*_2_, *λ*_3_, *p*_1_ and *p*_2_ may be written as:

$$\pi_{1}\left(\phi \right)\propto 1;\;\lambda_{1},\;\lambda_{2},\;\lambda_{3}>0,\;p_{1},\;p_{2}\geq 0,\;p_{1}+p_{2}\leq 1.$$ (9)

The joint posterior distribution of parameters *λ*_1_, *λ*_2_, *λ*_3_, *p*_1_ and *p*_2_ given data **y**, using the UP is given by (see S1 File):

$$g_{1}\left(\phi |y\right)=\frac{L\left(\phi |y\right)\pi_{1}\left(\phi \right)}{\int_{\phi }L\left(\phi |y\right)\pi_{1}\left(\phi \right)d\phi }$$ (10)

$$g_{1}\left(\phi |y\right)=\frac{\sum_{i=0}^{n-r}\sum_{j=0}^{i}\left(\begin{array}{l} n-r\\ \;i \end{array}\right)\left(\begin{array}{l} i\\ \text{j} \end{array}\right)\exp\left(-\frac{B_{11}}{\lambda_{1}^{2}}\right)\exp\left(-\frac{B_{21}}{\lambda_{2}^{2}}\right)\exp\left(-\frac{B_{31}}{\lambda_{3}^{2}}\right)p_{1}^{A_{01}-1}p_{2}^{B_{01}-1}{\left(1-p_{1}-p_{2}\right)}^{C_{01}-1}}{\Omega_{1}\;\lambda_{1}^{2A_{11}+1}\lambda_{2}^{2A_{21}+1}\lambda_{3}^{2A_{31}+1}},$$ (11)

where $A_{11}=r_{1}-\frac{1}{2}$, $A_{21}=r_{2}-\frac{1}{2}$, $A_{31}=r_{3}-\frac{1}{2}$, $B_{11}=\left(n-r-i\right)\frac{t^{2}}{2}+\frac{1}{2}\sum_{k=1}^{r_{1}}y_{1k}^{2}$, $B_{21}=\left(i-j\right)\frac{t^{2}}{2}+\frac{1}{2}\sum_{k=1}^{r_{2}}y_{2k}^{2}$, $B_{31}=j\frac{t^{2}}{2}+\frac{1}{2}\sum_{k=1}^{r_{3}}y_{3k}^{2}$, *A*_01_ = *n*−*r*−*i*+*r*_1_+1, *B*_01_ = *i*−*j*+*r*_2_+1, *C*_01_ = *j*+*r*_3_+1, $\Omega_{1}=\frac{1}{8}\Gamma \left(A_{11}\right)\Gamma \left(A_{21}\right)\Gamma \left(A_{31}\right)\sum_{i=0}^{n-r}\sum_{j=0}^{i}\left(\begin{array}{l} n-r\\ \;i \end{array}\right)\left(\begin{array}{l} i\\ \text{j} \end{array}\right)B\left(A_{01},B_{01},C_{01}\right){\text{B}}_{11}^{-A_{11}}{\text{B}}_{21}^{-A_{21}}{\text{B}}_{31}^{-A_{31}}$.

### 3.3 The posterior distribution usingthe Jeffreys’ prior

According to Jeffreys [27, 28], Bernardo [29] and Berger [30], the Jeffreys’ prior (JP) for *λ*_*m*_ (*m* = 1, 2, 3) is defined as $p\left(\lambda_{m}\right)\propto \sqrt{\left|I\left(\lambda_{m}\right)\right|}$, where $I\left(\lambda_{m}\right)=-E\left[\frac{\partial^{2}f\left(y;\lambda_{m}\right)}{\partial \lambda_{m}^{2}}\right]$ is the Fisher’s information matrix. It is interesting to note that the JP for proportion parameters *p*_1_ and *p*_2_ cannot be assumed under the current settings. Therefore, again, the uniform distribution over the interval (0,1) is assumed for both the *p*_1_ and *p*_2_, i.e., *p*_1_ ∼ (0,1) and *p*_2_ ∼ (0,1). Under the assumption of independence of all the parameters, the joint prior distribution of parameters *λ*_1_, *λ*_2_, *λ*_3_, *p*_1_ and *p*_2_ is given by:

$$\pi_{2}\left(\phi \right)\propto \frac{1}{\lambda_{1}\lambda_{2}\lambda_{3}},\;\lambda_{1},\;\lambda_{2},\;\lambda_{3}>0,\;p_{1},\;p_{2}\geq 0,\;p_{1}+p_{2}\leq 1.$$ (12)

Now, the joint posterior distribution of parameters *λ*_1_, *λ*_2_, *λ*_3_, *p*_1_ and *p*_2_ given data **y**, is given by (see S1 File):

$$g_{2}\left(\phi |y\right)=\frac{L\left(\phi |y\right)\pi_{2}\left(\phi \right)}{\int_{\phi }L\left(\phi |y\right)\pi_{2}\left(\phi \right)d\phi }$$ (13)

$$g_{2}\left(\phi |y\right)=\frac{\sum_{i=0}^{n-r}\sum_{j=0}^{i}\left(\begin{array}{l} n-r\\ \;i \end{array}\right)\left(\begin{array}{l} i\\ \text{j} \end{array}\right)\exp\left(-\frac{B_{12}}{\lambda_{1}^{2}}\right)\exp\left(-\frac{B_{22}}{\lambda_{2}^{2}}\right)\exp\left(-\frac{B_{32}}{\lambda_{3}^{2}}\right)p_{1}^{A_{02}-1}p_{2}^{B_{02}-1}{\left(1-p_{1}-p_{2}\right)}^{C_{02}-1}}{\Omega_{2}\;\lambda_{1}^{2A_{12}+1}\lambda_{2}^{2A_{22}+1}\lambda_{3}^{2A_{32}+1}},$$ (14)

where *A*_12_ = *r*_1_, *A*_22_ = *r*_2_, *A*_32_ = *r*_3_, $B_{12}=\left(n-r-i\right)\frac{t^{2}}{2}+\frac{1}{2}\sum_{k=1}^{r_{1}}y_{1k}^{2}$, $B_{22}=\left(i-j\right)\frac{t^{2}}{2}+\frac{1}{2}\sum_{k=1}^{r_{2}}y_{2k}^{2}$, $B_{32}=j\frac{t^{2}}{2}+\frac{1}{2}\sum_{k=1}^{r_{3}}y_{3k}^{2}$, *A*_02_ = *n*−*r*−*i*+*r*_1_+1, *B*_02_ = *i*−*j*+*r*_2_+1, *C*_02_ = *j*+*r*_3_+1, $\Omega_{2}=\frac{1}{8}\Gamma \left(A_{12}\right)\Gamma \left(A_{22}\right)\Gamma \left(A_{32}\right)\sum_{i=0}^{n-r}\sum_{j=0}^{i}\left(\begin{array}{l} n-r\\ \;i \end{array}\right)\left(\begin{array}{l} i\\ \text{j} \end{array}\right)B\left(A_{02},B_{02},C_{02}\right){\text{B}}_{12}^{-A_{12}}{\text{B}}_{22}^{-A_{22}}{\text{B}}_{32}^{-A_{32}}$.

### 3.4 The posterior distribution using the inverted chi-square prior

As an informative prior, we take inverted chi-square prior (ICP) for component parameters *λ*_1_, *λ*_2_, *λ*_3_ and bivariate beta prior for proportion parameters *p*_1_, *p*_2_. Symbolically, it can be written as: *λ*_1_ ∼ *IC*(*a*_1_,*b*_1_), *λ*_2_ ∼ *IC*(*a*_2_,*b*_2_), *λ*_3_ ∼ *IC*(*a*_3_,*b*_3_), and *p*_1_, *p*_2_ ∼ *Bi*var*iate Beta*(*a*,*b*,*c*). Again, assuming the independence of parameters, the joint prior distribution of parameters *λ*_1_, *λ*_2_, *λ*_3_, *p*_1_ and *p*_2_ is given by:

$$\pi_{3}\left(\phi \right)\propto \lambda_{1}^{-\left(a_{1}+1\right)}\exp\left(-\frac{b_{1}}{2\lambda_{1}^{2}}\right)\lambda_{2}^{-\left(a_{2}+1\right)}\exp\left(-\frac{b_{2}}{2\lambda_{2}^{2}}\right)\lambda_{3}^{-\left(a_{3}+1\right)}\exp\left(-\frac{b_{3}}{2\lambda_{3}^{2}}\right)p_{1}^{a-1}p_{2}^{b-1}{\left(1-p_{1}-p_{2}\right)}^{c-1}.$$ (15)

The joint posterior distribution of parameters *λ*_1_, *λ*_2_, *λ*_3_, *p*_1_ and *p*_2_ given data **y** is given by (see S1 File):

$$g_{3}\left(\phi |y\right)=\frac{L\left(\phi |y\right)\pi_{3}\left(\phi \right)}{\int_{\phi }L\left(\phi |y\right)\pi_{3}\left(\phi \right)d\phi }$$ (16)

$$g_{3}\left(\phi |y\right)=\frac{\sum_{i=0}^{n-r}\sum_{j=0}^{i}\left(\begin{array}{l} n-r\\ \;i \end{array}\right)\left(\begin{array}{l} i\\ \text{j} \end{array}\right)\exp\left(-\frac{B_{13}}{\lambda_{1}^{2}}\right)\exp\left(-\frac{B_{23}}{\lambda_{2}^{2}}\right)\exp\left(-\frac{B_{33}}{\lambda_{3}^{2}}\right)p_{1}^{A_{03}-1}p_{2}^{B_{03}-1}{\left(1-p_{1}-p_{2}\right)}^{C_{03}-1}}{\Omega_{3}\;\lambda_{1}^{2A_{13}+1}\lambda_{2}^{2A_{23}+1}\lambda_{3}^{2A_{33}+1}},$$ (17)

where $A_{13}=r_{1}+\frac{a_{1}}{2}$, $A_{23}=r_{2}+\frac{a_{2}}{2}$, $A_{33}=r_{3}+\frac{a_{3}}{2}$, $B_{13}=\left(n-r-i\right)\frac{t^{2}}{2}+\frac{1}{2}\sum_{k=1}^{r_{1}}y_{1k}^{2}+\frac{b_{1}}{2}$, $B_{23}=\left(i-j\right)\frac{t^{2}}{2}+\frac{1}{2}\sum_{k=1}^{r_{2}}y_{2k}^{2}+\frac{b_{2}}{2}$, $B_{33}=j\frac{t^{2}}{2}+\frac{1}{2}\sum_{k=1}^{r_{3}}y_{3k}^{2}+\frac{b_{3}}{2}$, *A*_03_ = *n*−*r*−*i*+*r*_1_+*a*, *B*_03_ = *i*−*j*+*r*_2_+*b*, *C*_03_ = *j*+*r*_3_+*c*, $\Omega_{3}=\frac{1}{8}\Gamma \left(A_{13}\right)\Gamma \left(A_{23}\right)\Gamma \left(A_{33}\right)\sum_{i=0}^{n-r}\sum_{j=0}^{i}\left(\begin{array}{l} n-r\\ \;i \end{array}\right)\left(\begin{array}{l} i\\ \text{j} \end{array}\right)B\left(A_{03},B_{03},C_{03}\right){\text{B}}_{13}^{-A_{13}}{\text{B}}_{23}^{-A_{23}}{\text{B}}_{33}^{-A_{33}}$.

### 3.5 The posterior distribution using the square root inverted gamma prior

Now, we assume the square root inverted gamma prior (SRIGP)as an informative prior for component parameters *λ*_1_, *λ*_2_, *λ*_3_, i.e., *λ*_1_ ∼ *SRIG*(*a*_1_,*b*_1_), *λ*_2_ ∼ *SRIG*(*a*_2_,*b*_2_) and *λ*_3_ ∼ *SRIG*(*a*_3_,*b*_3_), and abivariate beta prioras an informative prior for proportion parameters *p*_1_, *p*_2_, i.e., *p*_1_, *p*_2_ ∼ *Bi*var*iate Beta*(*a*,*b*,*c*). So, assuming the independence of parameters, the joint prior distribution of parameters *λ*_1_, *λ*_2_, *λ*_3_, *p*_1_ and *p*_2_ is given by:

$$\pi_{4}\left(\phi \right)\propto \lambda_{1}^{-\left(2a_{1}+1\right)}\exp\left(-\frac{b_{1}}{\lambda_{1}^{2}}\right)\lambda_{2}^{-\left(2a_{2}+1\right)}\exp\left(-\frac{b_{2}}{\lambda_{2}^{2}}\right)\lambda_{3}^{-\left(2a_{3}+1\right)}\exp\left(-\frac{b_{3}}{\lambda_{3}^{2}}\right)p_{1}^{a-1}p_{2}^{b-1}{\left(1-p_{1}-p_{2}\right)}^{c-1}.$$ (18)

In this case, the joint posterior distribution of parameters *λ*_1_, *λ*_2_, *λ*_3_, *p*_1_ and *p*_2_ given data **y** is given by (see S1 File):

$$g_{4}\left(\phi |y\right)=\frac{L\left(\phi |y\right)\pi_{4}\left(\phi \right)}{\int_{\phi }L\left(\phi |y\right)\pi_{4}\left(\phi \right)d\phi }$$ (19)

$$g_{4}\left(\phi |y\right)=\frac{\sum_{i=0}^{n-r}\sum_{j=0}^{i}\left(\begin{array}{l} n-r\\ \;i \end{array}\right)\left(\begin{array}{l} i\\ \text{j} \end{array}\right)\exp\left(-\frac{B_{14}}{\lambda_{1}^{2}}\right)\exp\left(-\frac{B_{24}}{\lambda_{2}^{2}}\right)\exp\left(-\frac{B_{34}}{\lambda_{3}^{2}}\right)p_{1}^{A_{04}-1}p_{2}^{B_{04}-1}{\left(1-p_{1}-p_{2}\right)}^{C_{04}-1}}{\Omega_{4}\;\lambda_{1}^{2A_{14}+1}\lambda_{2}^{2A_{24}+1}\lambda_{3}^{2A_{34}+1}},$$ (20)

where *A*_14_ = *r*_1_+*a*_1_, *A*_24_ = *r*_2_+*a*_2_, *A*_34_ = *r*_3_+*a*_3_, $B_{14}=\left(n-r-i\right)\frac{t^{2}}{2}+\frac{1}{2}\sum_{k=1}^{r_{1}}y_{1k}^{2}+b_{1}$, $B_{24}=\left(i-j\right)\frac{t^{2}}{2}+\frac{1}{2}\sum_{k=1}^{r_{2}}y_{2k}^{2}+b_{2}$, $B_{34}=j\frac{t^{2}}{2}+\frac{1}{2}\sum_{k=1}^{r_{3}}y_{3k}^{2}+b_{3}$, *A*_04_ = *n*−*r*−*i*+*r*_1_+*a*, *B*_04_ = *i*−*j*+*r*_2_+*b*, *C*_04_ = *j*+*r*_3_+*c*, $\Omega_{4}=\frac{1}{8}\Gamma \left(A_{14}\right)\Gamma \left(A_{24}\right)\Gamma \left(A_{34}\right)\sum_{i=0}^{n-r}\sum_{j=0}^{i}\left(\begin{array}{l} n-r\\ \;i \end{array}\right)\left(\begin{array}{l} i\\ \text{j} \end{array}\right)B\left(A_{04},B_{04},C_{04}\right){\text{B}}_{14}^{-A_{14}}{\text{B}}_{24}^{-A_{24}}{\text{B}}_{34}^{-A_{34}}$.

## Elicitation of hyperparameters

Elicitation is a tool used to quantify a person’s prior belief and knowledge. In Bayesian perspective, elicitation most often arises as a method of specifying the prior distribution of the random parameter(s). Elicitation is simply the quantification of prior knowledge about the random parameter(s) so that this can then be combined with the likelihood to obtain posterior distribution for further statistical analysis. Elicitation has remained a challenging problem for the statistician.Authors who have discussed this problem include Kadane et al. [31], Gavasakar [32], Al-Awadhi and Gartwaite [33], Aslam [34], Hahn [35] and Saleem and Aslam [18]. In this study, we adopted prior predictive method based on predictive probabilities given by Aslam [34].

### 4.1 Elicitation of hyperparameters using the ICP

For eliciting the hyperparameters, prior predictive distribution (PPD) is used. The PPD using the ICP for a random variable *Y* is defined as:

$$p\left(y\right)=\int_{\phi }f\left(y|\phi \right)\pi_{3}\left(\phi \right)d\phi$$ (21)

On substituting (4) and (15) in (21) and then simplifying, we get:

$$p\left(y\right)=\frac{1}{\left(a+b+c\right)}\left(\frac{aa_{1}b_{1}^{\frac{a_{1}}{2}}y}{{\left(b_{1}+y^{2}\right)}^{\frac{a_{1}}{2}+1}}+\frac{ba_{2}b_{2}^{\frac{a_{2}}{2}}y}{{\left(b_{2}+y^{2}\right)}^{\frac{a_{2}}{2}+1}}+\frac{ca_{3}b_{3}^{\frac{a_{3}}{2}}y}{{\left(b_{3}+y^{2}\right)}^{\frac{a_{3}}{2}+1}}\right).$$ (22)

Using the prior predictive distribution given in (22), we consider nine intervals (0, 0.5), (0.5, 1), (1, 1.5), (1.5, 2), (2, 2.5), (2.5, 3), (3, 3.5), (3.5, 4) and (4, 4.5) with respective probabilities 0.12, 0.26, 0.24, 0.15, 0.10, 0.05, 0.03, 0.02 and 0.01 as an expert’s belief about these intervals.

Using (22), following nine equations in (23) are solved simultaneously in Mathematica package for eliciting the hyperparameters *a*_1_, *b*_1_, *a*_2_, *b*_2_, *a*_3_, *b*_3_, *a*, *b* and *c*.

$$\begin{array}{l} \int_{0}^{0.5}p\left(y\right)\;dy=0.12;\;\int_{0.5}^{1}p\left(y\right)\;dy=0.26;\;\int_{1}^{1.5}p\left(y\right)\;dy=0.24;\\ \int_{1.5}^{2}p\left(y\right)\;dy=0.15;\;\int_{2}^{2.5}p\left(y\right)\;dy=0.10;\;\int_{2.5}^{3}p\left(y\right)\;dy=0.05;\\ \int_{3}^{3.5}p\left(y\right)\;dy=0.03;\;\int_{3.5}^{4}p\left(y\right)\;dy=0.02;\;\int_{4}^{4.5}p\left(y\right)\;dy=0.01; \end{array}\}.$$ (23)

The elicited values of the hyperparameters *a*_1_, *b*_1_, *a*_2_, *b*_2_, *a*_3_, *b*_3_, *a*, *b* and *c* are obtained as 5.88796, 5.67093, 5.4940, 5.28366, 4.90644, 4.68736, 3.46665, 4.68959 and 4.30064, respectively.

### 4.2 Elicitation of hyperparameters using the SRIGP

The PPD using SRIGP for a random variable *Y* is given by:

$$p\left(y\right)=\int_{\phi }f\left(y|\phi \right)\pi_{4}\left(\phi \right)d\phi$$ (24)

Using (4), (18) and (24), we get:

$$p\left(y\right)=\frac{1}{\left(a+b+c\right)}\left(\frac{aa_{1}b_{1}^{a_{1}}y}{{\left(b_{1}+\frac{y^{2}}{2}\right)}^{a_{1}+1}}+\frac{ba_{2}b_{2}^{a_{2}}y}{{\left(b_{2}+\frac{y^{2}}{2}\right)}^{a_{2}+1}}+\frac{ca_{3}b_{3}^{a_{3}}y}{{\left(b_{3}+\frac{y^{2}}{2}\right)}^{a_{3}+1}}\right).$$ (25)

Through the above criteria as defined in Subsection 4.1, the values of the hyperparameters *a*_1_, *b*_1_, *a*_2_, *b*_2_, *a*_3_, *b*_3_, *a*, *b* and *c* are now obtained as 5.74419, 4.97886, 5.65643, 5.43122, 4.93333, 4.93038, 11.8838, 6.41829 and 7.0491, respectively.

## Bayes estimators and posterior risks using the UP, the JP, the ICPand the SRIGPunder SELF, PLF and DLF

If $\hat{d}$ is a Bayes estimator then $\rho \left(\hat{d}\right)$ is called posterior risk and is defined as: $\rho \left(\hat{d}\right)=E_{\lambda |y}\left\{L\left(\lambda ,\hat{d}\right)\right\}$. Our purpose, in this study, is to look for efficient Bayes estimators of the different parameters. For this purpose, three different loss functions, namely, SELF, PLF and DLF are used to obtain the Bayes estimators and their posterior risks. The SELF, defined as *L*(*λ*,*d*) = (*λ* – *d*)^2^, was introduced by Legendre [36] to develop the least square theory. Norstrom [37] discussed an asymmetric PLF and also introduced a special case of general class of PLFs, which is defined as $L\left(\lambda ,d\right)=\frac{{\left(\lambda -d\right)}^{2}}{d}$. The PLF approaches infinitely close to the origin to avert underestimation, so yielding conventional estimators when underestimation may lead to grave results. The DLF is presented by DeGroot [38] and is defined as $L\left(\lambda ,d\right)={\left(\frac{\lambda -d}{d}\right)}^{2}$.

For a given prior, the Bayes estimator and posterior risk under SELF are calculated as: $\hat{d}=E_{\lambda |y}\left(\lambda \right)$ and $\rho \left(\hat{d}\right)=E_{\lambda |y}\left(\lambda^{2}\right)-{\left\{E_{\lambda |y}\left(\lambda \right)\right\}}^{2}$, respectively. Similarly, the Bayes estimators and posterior risks with PLF and DLF are given by: $\hat{d}={\left\{E_{\lambda |y}\left(\lambda^{2}\right)\right\}}^{\frac{1}{2}}$, $\rho \left(\hat{d}\right)=2{\left\{E_{\lambda |y}\left(\lambda^{2}\right)\right\}}^{\frac{1}{2}}-2E_{\lambda |y}\left(\lambda \right)$, and $\hat{d}=\frac{E_{\lambda |y}\left(\lambda^{2}\right)}{E_{\lambda |y}\left(\lambda \right)}$, $\rho \left(\hat{d}\right)=1-\frac{{\left\{E_{\lambda |y}\left(\lambda \right)\right\}}^{2}}{E_{\lambda |y}\left(\lambda^{2}\right)}$, respectively. The Bayes estimators and posterior risks using the UP, the JP, the ICP and the SRIGP for the parameters *λ*_1_, *λ*_2_, *λ*_3_, *p*_1_ and *p*_2_ under SELF, PLF and DLF are obtained as:

$${\hat{\lambda }}_{1v}=\frac{\Gamma \left(A_{1v}-0.5\right)\Gamma \left(A_{2v}\right)\Gamma \left(A_{3v}\right)}{8\Omega_{v}}\sum_{i=0}^{n-r}\sum_{j=0}^{i}\left(\begin{array}{l} n-r\\ \;i \end{array}\right)\left(\begin{array}{l} i\\ \text{j} \end{array}\right){\text{B}}_{1v}^{-\left(A_{1v}-0.5\right)}{\text{B}}_{2v}^{-A_{2v}}{\text{B}}_{3v}^{-A_{3v}}B\left(A_{0v},C_{0v}\right)B\left(B_{0v},A_{0v}+C_{0v}\right)$$ (26)

$${\hat{\lambda }}_{2v}=\frac{\Gamma \left(A_{1v}\right)\Gamma \left(A_{2v}-0.5\right)\Gamma \left(A_{3v}\right)}{8\Omega_{v}}\sum_{i=0}^{n-r}\sum_{j=0}^{i}\left(\begin{array}{l} n-r\\ \;i \end{array}\right)\left(\begin{array}{l} i\\ \text{j} \end{array}\right){\text{B}}_{1v}^{-A_{1v}}{\text{B}}_{2v}^{-\left(A_{2v}-0.5\right)}{\text{B}}_{3v}^{-A_{3v}}B\left(A_{0v},C_{0v}\right)B\left(B_{0v},A_{0v}+C_{0v}\right)$$ (27)

$${\hat{\lambda }}_{3v}=\frac{\Gamma \left(A_{1v}\right)\Gamma \left(A_{2v}\right)\Gamma \left(A_{3v}-0.5\right)}{8\Omega_{v}}\sum_{i=0}^{n-r}\sum_{j=0}^{i}\left(\begin{array}{l} n-r\\ \;i \end{array}\right)\left(\begin{array}{l} i\\ \text{j} \end{array}\right){\text{B}}_{1v}^{-A_{1v}}{\text{B}}_{2v}^{-A_{2v}}{\text{B}}_{3v}^{-\left(A_{3v}-0.5\right)}B\left(A_{0v},C_{0v}\right)B\left(B_{0v},A_{0v}+C_{0v}\right)$$ (28)

$${\hat{p}}_{1v}=\frac{\Gamma \left(A_{1v}\right)\Gamma \left(A_{2v}\right)\Gamma \left(A_{3v}\right)}{8\Omega_{v}}\sum_{i=0}^{n-r}\sum_{j=0}^{i}\left(\begin{array}{l} n-r\\ \;i \end{array}\right)\left(\begin{array}{l} i\\ \text{j} \end{array}\right){\text{B}}_{1v}^{-A_{1v}}{\text{B}}_{2v}^{-A_{2v}}{\text{B}}_{3v}^{-A_{3v}}B\left(B_{0v},C_{0v}\right)B\left(A_{0v}+1,B_{0v}+C_{0v}\right)$$ (29)

$${\hat{p}}_{2v}=\frac{\Gamma \left(A_{1v}\right)\Gamma \left(A_{2v}\right)\Gamma \left(A_{3v}\right)}{8\Omega_{v}}\sum_{i=0}^{n-r}\sum_{j=0}^{i}\left(\begin{array}{l} n-r\\ \;i \end{array}\right)\left(\begin{array}{l} i\\ \text{j} \end{array}\right){\text{B}}_{1v}^{-A_{1v}}{\text{B}}_{2v}^{-A_{2v}}{\text{B}}_{3v}^{-A_{3v}}B\left(A_{0v},C_{0v}\right)B\left(B_{0v}+1,A_{0v}+C_{0v}\right)$$ (30)

$$\rho \left({\hat{\lambda }}_{1v}\right)=\frac{\Gamma \left(A_{1v}-1\right)\Gamma \left(A_{2v}\right)\Gamma \left(A_{3v}\right)}{8\Omega_{v}}\sum_{i=0}^{n-r}\sum_{j=0}^{i}\left(\begin{array}{l} n-r\\ \;i \end{array}\right)\left(\begin{array}{l} i\\ \text{j} \end{array}\right){\text{B}}_{1v}^{-\left(A_{1v}-1\right)}{\text{B}}_{2v}^{-A_{2v}}{\text{B}}_{3v}^{-A_{3v}}B\left(A_{0v},C_{0v}\right)B\left(B_{0v},A_{0v}+C_{0v}\right)-{\left({\hat{\lambda }}_{1v}\right)}^{2}$$ (31)

$$\rho \left({\hat{\lambda }}_{2v}\right)=\frac{\Gamma \left(A_{1v}\right)\Gamma \left(A_{2v}-1\right)\Gamma \left(A_{3v}\right)}{8\Omega_{v}}\sum_{i=0}^{n-r}\sum_{j=0}^{i}\left(\begin{array}{l} n-r\\ \;i \end{array}\right)\left(\begin{array}{l} i\\ \text{j} \end{array}\right){\text{B}}_{1v}^{-A_{1v}}{\text{B}}_{2v}^{-\left(A_{2v}-1\right)}{\text{B}}_{3v}^{-A_{3v}}B\left(A_{0v},C_{0v}\right)B\left(B_{0v},A_{0v}+C_{0v}\right)-{\left({\hat{\lambda }}_{2v}\right)}^{2}$$ (32)

$$\rho \left({\hat{\lambda }}_{3v}\right)=\frac{\Gamma \left(A_{1v}\right)\Gamma \left(A_{2v}\right)\Gamma \left(A_{3v}-1\right)}{8\Omega_{v}}\sum_{i=0}^{n-r}\sum_{j=0}^{i}\left(\begin{array}{l} n-r\\ \;i \end{array}\right)\left(\begin{array}{l} i\\ \text{j} \end{array}\right){\text{B}}_{1v}^{-A_{1v}}{\text{B}}_{2v}^{-A_{2v}}{\text{B}}_{3v}^{-\left(A_{3v}-1\right)}B\left(A_{0v},C_{0v}\right)B\left(B_{0v},A_{0v}+C_{0v}\right)-{\left({\hat{\lambda }}_{3v}\right)}^{2}$$ (33)

$$\rho \left({\hat{p}}_{1v}\right)=\frac{\Gamma \left(A_{1v}\right)\Gamma \left(A_{2v}\right)\Gamma \left(A_{3v}\right)}{8\Omega_{v}}\sum_{i=0}^{n-r}\sum_{j=0}^{i}\left(\begin{array}{l} n-r\\ \;i \end{array}\right)\left(\begin{array}{l} i\\ \text{j} \end{array}\right){\text{B}}_{1v}^{-A_{1v}}{\text{B}}_{2v}^{-A_{2v}}{\text{B}}_{3v}^{-A_{3v}}B\left(B_{0v},C_{0v}\right)B\left(A_{0v}+2,B_{0v}+C_{0v}\right)-{\left({\hat{p}}_{1v}\right)}^{2}$$ (34)

$$\rho \left({\hat{p}}_{2v}\right)=\frac{\Gamma \left(A_{1v}\right)\Gamma \left(A_{2v}\right)\Gamma \left(A_{3v}\right)}{8\Omega_{v}}\sum_{i=0}^{n-r}\sum_{j=0}^{i}\left(\begin{array}{l} n-r\\ \;i \end{array}\right)\left(\begin{array}{l} i\\ \text{j} \end{array}\right){\text{B}}_{1v}^{-A_{1v}}{\text{B}}_{2v}^{-A_{2v}}{\text{B}}_{3v}^{-A_{3v}}B\left(A_{0v},C_{0v}\right)B\left(B_{0v}+2,A_{0v}+C_{0v}\right)-{\left({\hat{p}}_{2v}\right)}^{2},$$ (35)

where *v* = 1 for the UP, *v* = 2 for the JP, *v* = 3 for the ICP and *v* = 4 for the SRIGP. The Bayes estimators and posterior risks using the UP, the JP, the ICP and the SRIGP under PLF and DLF can also be derived in similar way and are presented as supporting information in S1 File.

## Limiting expressions

When test termination time *t*→∞, uncensored observations *r* tends to sample size *n* and *r*_*l*_ tends to *n*_*l*_, *l* = 1,2,3. Consequently, all the observations which are censored become uncensored and the information contained in the sample is increased. As a result, the posterior risks of the Bayes estimatorsdiminish and efficiency of the Bayes estimators is increased because all the observations are incorporated in sample. The limiting expressions for the Bayes estimators and posterior risks using the UP, the JP, the ICP and the SRIGP under SELF are given in Tables A-D in S2 File. The limiting expressions for the Bayes estimators and posterior risks using the UP, the JP, the ICP and the SRIGP under PLF and DLF can also be derived in similar way. These limiting expressions can be used in case of uncensored sampling schemes.

## Simulation study

To know the performance of Bayes estimatorsunder different priors, loss functions, sample sizes and test termination times. Samples of sizes, *n* = 50, 100, 200, 500 are generated from a 3-component mixture of Rayleigh distributions with different set of parametric values *λ*_1_, *λ*_2_, *λ*_3_, *p*_1_ and *p*_2_ fixed as (*λ*_1_, *λ*_2_, *λ*_3_, *p*_1_, *p*_2_) = {(14, 12, 10, 0.5, 0.3), (16, 14, 12, 0.5, 0.3), (11, 13, 15, 0.3, 0.5)}.

For a fixed sample size, test termination time and set of parameters, the *p*_1_ *n* (*p*_2_ *n*,(1 − *p*_1_ − *p*_2_)*n*) observations are randomly taken from first (second, third) component density.The observations which are greater than a fixed *t* are declared as censored observations. For each *t*, only failures are identified either as member of subpopulation-I or subpopulation-II or subpopulation-III…Based on such sample, the Bayes estimates (BEs) and posterior risks (PRs)are computed using the UP, the JP, the CIP and the SRIGP under SELF, PLF and DLF. In order to evaluate the impact of test termination time on Bayes estimators, the type-I right censoring scheme is used for fixed test termination times *t* = 25 and 30. All the above procedure is repeated 1000 times using Mathematica software. The results are then averaged over the 1000 samples and are arranged in S1–S12 Tables.

From S1–S12 Tables, it can be seen that the extent of over-estimation (under-estimation) of thecomponent and proportion parameters (through Bayes estimators)using all considered priors and loss functions is greaterfor small sample size (test termination time)as compared to large sample size (test termination time) at different test termination times (sample sizes).Similarly,the extent of over-estimation (under-estimation) of component and proportion parameters is lesserfor smaller values of component parameters as compared to larger values of component parameters atvaryingtest termination times and sample sizes. It is observed that difference of the BEs from assumed parameters reduce to zero with an increase in sample size for different test termination times.The same observation can be made with larger test termination time as compared to smaller test termination time for varying sample sizes.

It is observed that the PRs of Bayes estimatorsusingthe different priors and loss functions reduce with an increase in samplesize at different test termination times.For smallertest termination time, the PRs of Bayes estimators are larger than the PRs for large test termination time irrespective of the prior, loss function and sample size. Also, the PRs of Bayes estimators are smaller (larger) for smaller (larger) component parametric values for each sample size and test termination time considered in the simulation study.

As far as the problem of selecting a suitable prior is concerned, it can be seen that SRIGP emerges as the best prior amongst the different non-informative and informative priors considered in this study. On the other hand, the DLF is observed performing better than PLF and SELF for estimating component parameters, whereas, for estimating the proportion parameters, SELF is observed superior to PLF and DLF. It is to be noted that selection of best prior (loss function) for a given loss function (prior) is made based on PRs associated with it. Also, the selection of best prior and loss function does not depend on sample size and test termination time.

## Real data application

The real mixture data, $z=\left(z_{11},\;z_{12},\ldots ,\;z_{1r_{1}},\;z_{21},\;z_{22},\ldots ,\;z_{2r_{2}},\;z_{31},\;z_{32},\ldots ,\;z_{3r_{3}}\right)$, are taken from Davis [39]. These data represent hours to failure of a V805 Transmitter Tube, a Transmitter Tube and a V600 Indicator Tube used in aircraft radar sets. Davis [39] showed that the data **z** can be modeled by a mixture of exponential distributions. The transformation $y=\sqrt{2z}$ of an exponential random data (**z**) yields the Rayleigh random data (**y**). This transformation allows us to use the Davis mixture data for applying the proposed Bayesian analysis. To have a type-I right censored data we fix *t* = 600 hours. The tests are conducted 1340 times. Thus, we have a type-I right censored data at *t* = 600 hours on *n* = 1340 radar sets. The data summary required to evaluate the BEs and PRs is given by: $\sum_{k=1}^{r_{1}}y_{1k}^{2}=2\sum_{k=1}^{r_{1}}z_{1k}=268160$, $\sum_{k=1}^{r_{2}}y_{2k}^{2}=2\sum_{k=1}^{r_{2}}z_{2k}=100750$, $\sum_{k=1}^{r_{3}}y_{3k}^{2}=2\sum_{k=1}^{r_{3}}z_{3k}=32500$, *n* = 1340, *r*_1_ = 866, *r*_2_ = 337, *r*_3_ = 83, *r* = *r*_1_+*r*_2_+*r*_3_ = 1286, *n*−*r* = 54.

The BEs and the PRs using the UP, the JP, the ICP and the SRIGP under SELF, PLF and DLF are presented in S13 Table.

From S13 Table, it is observed that the results based on the real data are compatible with simulation results.The results about the best prior and the best loss function are also the same as we have discussed in the Section 7.

## Concluding remarks

In this study, we have considered the Bayesian analysis of3-componenten mixture of Rayleigh distributionsusing the non-informative (uniform and Jeffreys’) and the informative (IC and SRIG) priors under SELF, PLF and DLFto model lifetimes of objects. We conducted a comprehensive simulation and real life study to judge the relative performance of the Bayes estimators and also to deal with the problems of selecting the priors and loss functions at different sample sizes and test termination times. From simulated results, we observed that an increase in sample size or test termination time provides improved Bayes estimators. The extent of over-estimation (under-estimation) of the Bayes estimators is quite larger (smaller)for relatively smaller(larger) sample sizes (test termination times) at different test termination times (sample sizes). Furthermore, as sample size (test termination time) increases (decreases) the PRs of Bayes estimators decrease (increase) for a fixed test termination time (sample size). However, the PRs of Bayes estimators are large when component parameters are relatively larger and vice versa.Also, the DLF (SELF) is observed as a suitable choice for estimating component (proportion) parameters.Finally, we conclude that the SRIGP is more suitable prior under DLF for estimating the component parameters. In case, when SELFis used, the SRIGP is preferablepriorfor proportion parameters. Moreover, the same pattern is observed for the JP when only non-informative priors (UP and JP) are considered.

## Supporting Information

S1 File Derivation of likelihood function, posterior distributions, Bayes estimators and their risks under different priors and loss functions. (DOC)

S2 File Limiting expressions for the Bayes estimators. (DOC)

S1 Table The BEs and the PRs using the UP with *λ*_1_ = 14, *λ*_2_ = 12, *λ*_3_ = 10, *p*_1_ = 0.5, *p*_2_ = 0.3 and *t* = 25, 30. (DOCX)

S2 Table The BEs and the PRs using the JP with *λ*_1_ = 14, *λ*_2_ = 12, *λ*_3_ = 10, *p*_1_ = 0.5, *p*_2_ = 0.3 and *t* = 25, 30. (DOCX)

S3 Table The BEs and the PRs using the UP with *λ*_1_ = 16, *λ*_2_ = 14, *λ*_3_ = 12, *p*_1_ = 0.5, *p*_2_ = 0.3 and *t* = 25, 30. (DOCX)

S4 Table The BEs and the PRs using the JP with *λ*_1_ = 16, *λ*_2_ = 14, *λ*_3_ = 12, *p*_1_ = 0.5, *p*_2_ = 0.3 and *t* = 25, 30. (DOCX)

S5 Table The BEs and the PRs using the ICP with *λ*_1_ = 14, *λ*_2_ = 12, *λ*_3_ = 10, *p*_1_ = 0.5, *p*_2_ = 0.3 and *t* = 25, 30. (DOCX)

S6 Table The BEs and the PRs using the SRIGP with *λ*_1_ = 14, *λ*_2_ = 12, *λ*_3_ = 10, *p*_1_ = 0.5, *p*_2_ = 0.3 and *t* = 25, 30. (DOCX)

S7 Table The BEs and the PRs using the ICP with *λ*_1_ = 16, *λ*_2_ = 14, *λ*_3_ = 12, *p*_1_ = 0.5, *p*_2_ = 0.3 and *t* = 25, 30. (DOCX)

S8 Table The BEs and the PRs using the SRIGP with *λ*_1_ = 16, *λ*_2_ = 14, *λ*_3_ = 12, *p*_1_ = 0.5, *p*_2_ = 0.3 and *t* = 25, 30. (DOCX)

S9 Table The BEs and the PRs using the UP with *λ*_1_ = 11, *λ*_2_ = 13, *λ*_3_ = 15, *p*_1_ = 0.3, *p*_2_ = 0.5 and *t* = 25, 30. (DOCX)

S10 Table The BEs and the PRs using the JP with *λ*_1_ = 11, *λ*_2_ = 13, *λ*_3_ = 15, *p*_1_ = 0.3, *p*_2_ = 0.5 and *t* = 25, 30. (DOCX)

S11 Table The BEs and the PRs using the ICP with *λ*_1_ = 11, *λ*_2_ = 13, *λ*_3_ = 15, *p*_1_ = 0.3, *p*_2_ = 0.5 and *t* = 25, 30. (DOCX)

S12 Table The BEs and the PRs using the SRIGP with *λ*_1_ = 11, *λ*_2_ = 13, *λ*_3_ = 15, *p*_1_ = 0.3, *p*_2_ = 0.5 and *t* = 25, 30. (DOCX)

S13 Table The BEs and the PRs using the UP, the JP, the ICP and the SRIGP under SELF, PLF and DLF. (DOCX)
