# Supplementary material for: A 3-Component Mixture of Rayleigh Distributions: Properties and Estimation in Bayesian Framework
Source: PLoS One. 2015 May 20;10(5):e0126183. doi: 10.1371/journal.pone.0126183 (PMC4439070; doi:10.1371/journal.pone.0126183)
Supplement: S1 File — (DOC) [file pone.0126183.s001.doc]

***Derivation of likelihood function***

For a 3-component mixture model, the likelihood function can be written as:

***Derivation of posterior distribution using the uniform prior***

where $A_{11}=r_{1}-\frac{1}{2}$, $A_{21}=r_{2}-\frac{1}{2}$, $A_{31}=r_{3}-\frac{1}{2}$, $B_{11}=\left(n-r-i\right)\frac{t^{2}}{2}+\frac{1}{2}\sum_{k=1}^{r_{1}} y_{1k}^{2}$, $B_{21}=\left(i-j\right)\frac{t^{2}}{2}+\frac{1}{2}\sum_{k=1}^{r_{2}} y_{2k}^{2}$,

$B_{31}=j\frac{t^{2}}{2}+\frac{1}{2}\sum_{k=1}^{r_{3}} y_{3k}^{2}$, $A_{01}=n-r-i+r_{1}+1$, $B_{01}=i-j+r_{2}+1$, $C_{01}=j+r_{3}+1$,

$\Omega_{1}=\frac{1}{8}\Gamma\left(A_{11}\right)\Gamma\left(A_{21}\right)\Gamma\left(A_{31}\right)\sum_{i=0}^{n-r} \sum_{j=0}^{i} \left(\begin{matrix} n-r \\ \text{ }i \end{matrix}\right)\left(\begin{matrix} i \\ \text{j} \end{matrix}\right)B\left(A_{01},B_{01},C_{01}\right)\text{B}_{11}^{-A_{11}}\text{B}_{21}^{-A_{21}}\text{B}_{31}^{-A_{31}}$.

***Derivation of posterior distribution using the Jeffreys’ prior***

where $A_{12}=r_{1}$, $A_{22}=r_{2}$, $A_{32}=r_{3}$, $B_{12}=\left(n-r-i\right)\frac{t^{2}}{2}+\frac{1}{2}\sum_{k=1}^{r_{1}} y_{1k}^{2}$, $B_{22}=\left(i-j\right)\frac{t^{2}}{2}+\frac{1}{2}\sum_{k=1}^{r_{2}} y_{2k}^{2}$,

$B_{32}=j\frac{t^{2}}{2}+\frac{1}{2}\sum_{k=1}^{r_{3}} y_{3k}^{2}$, $A_{02}=n-r-i+r_{1}+1$, $B_{02}=i-j+r_{2}+1$, $C_{02}=j+r_{3}+1$,

$\Omega_{2}=\frac{1}{8}\Gamma\left(A_{12}\right)\Gamma\left(A_{22}\right)\Gamma\left(A_{32}\right)\sum_{i=0}^{n-r} \sum_{j=0}^{i} \left(\begin{matrix} n-r \\ \text{ }i \end{matrix}\right)\left(\begin{matrix} i \\ \text{j} \end{matrix}\right)B\left(A_{02},B_{02},C_{02}\right)\text{B}_{12}^{-A_{12}}\text{B}_{22}^{-A_{22}}\text{B}_{32}^{-A_{32}}$.

***Derivation of posterior distribution using the inverted chi-square prior***

where $A_{13}=r_{1}+\frac{a_{1}}{2}$, $A_{23}=r_{2}+\frac{a_{2}}{2}$, $A_{33}=r_{3}+\frac{a_{3}}{2}$, $B_{13}=\left(n-r-i\right)\frac{t^{2}}{2}+\frac{1}{2}\sum_{k=1}^{r_{1}} y_{1k}^{2}+\frac{b_{1}}{2}$, $B_{23}=\left(i-j\right)\frac{t^{2}}{2}+\frac{1}{2}\sum_{k=1}^{r_{2}} y_{2k}^{2}+\frac{b_{2}}{2}$, $B_{33}=j\frac{t^{2}}{2}+\frac{1}{2}\sum_{k=1}^{r_{3}} y_{3k}^{2}+\frac{b_{3}}{2}$, $A_{03}=n-r-i+r_{1}+a$, $B_{03}=i-j+r_{2}+b$, $C_{03}=j+r_{3}+c$, $\Omega_{3}=\frac{1}{8}\Gamma\left(A_{13}\right)\Gamma\left(A_{23}\right)\Gamma\left(A_{33}\right)\sum_{i=0}^{n-r} \sum_{j=0}^{i} \left(\begin{matrix} n-r \\ \text{ }i \end{matrix}\right)\left(\begin{matrix} i \\ \text{j} \end{matrix}\right)B\left(A_{03},B_{03},C_{03}\right)\text{B}_{13}^{-A_{13}}\text{B}_{23}^{-A_{23}}\text{B}_{33}^{-A_{33}}$.

***Derivation of posterior distribution using the square root inverted gamma prior***

where $A_{14}=r_{1}+a_{1}$, $A_{24}=r_{2}+a_{2}$, $A_{34}=r_{3}+a_{3}$, $B_{14}=\left(n-r-i\right)\frac{t^{2}}{2}+\frac{1}{2}\sum_{k=1}^{r_{1}} y_{1k}^{2}+b_{1}$, $B_{24}=\left(i-j\right)\frac{t^{2}}{2}+\frac{1}{2}\sum_{k=1}^{r_{2}} y_{2k}^{2}+b_{2}$,

$B_{34}=j\frac{t^{2}}{2}+\frac{1}{2}\sum_{k=1}^{r_{3}} y_{3k}^{2}+b_{3}$,$A_{04}=n-r-i+r_{1}+a$, $B_{04}=i-j+r_{2}+b$, $C_{04}=j+r_{3}+c$,

$\Omega_{4}=\frac{1}{8}\Gamma\left(A_{14}\right)\Gamma\left(A_{24}\right)\Gamma\left(A_{34}\right)\sum_{i=0}^{n-r} \sum_{j=0}^{i} \left(\begin{matrix} n-r \\ \text{ }i \end{matrix}\right)\left(\begin{matrix} i \\ \text{j} \end{matrix}\right)B\left(A_{04},B_{04},C_{04}\right)\text{B}_{14}^{-A_{14}}\text{B}_{24}^{-A_{24}}\text{B}_{34}^{-A_{34}}$.

**Bayes estimators and posterior risks using the UP, the JP, the ICP and the SRIGP under PLF**

$$\hat{\lambda}_{1v}=\left\{\frac{\Gamma\left(A_{1v}-1\right)\Gamma\left(A_{2v}\right)\Gamma\left(A_{3v}\right)}{8\Omega_{v}}\sum_{i=0}^{n-r} \sum_{j=0}^{i} \left(\begin{matrix} n-r \\ \text{ }i \end{matrix}\right)\left(\begin{matrix} i \\ \text{j} \end{matrix}\right)\text{B}_{1v}^{-\left(A_{1v}-1\right)}\text{B}_{2v}^{-A_{2v}}\text{B}_{3v}^{-A_{3v}}B\left(A_{0v},C_{0v}\right)B\left(B_{0v},A_{0v}+C_{0v}\right)\right\}^{\frac{1}{2}}$$

$$\hat{\lambda}_{2v}=\left\{\frac{\Gamma\left(A_{1v}\right)\Gamma\left(A_{2v}-1\right)\Gamma\left(A_{3v}\right)}{8\Omega_{v}}\sum_{i=0}^{n-r} \sum_{j=0}^{i} \left(\begin{matrix} n-r \\ \text{ }i \end{matrix}\right)\left(\begin{matrix} i \\ \text{j} \end{matrix}\right)\text{B}_{1v}^{-A_{1v}}\text{B}_{2v}^{-\left(A_{2v}-1\right)}\text{B}_{3v}^{-A_{3v}}B\left(A_{0v},C_{0v}\right)B\left(B_{0v},A_{0v}+C_{0v}\right)\right\}^{\frac{1}{2}}$$

$$\hat{\lambda}_{3v}=\left\{\frac{\Gamma\left(A_{1v}\right)\Gamma\left(A_{2v}\right)\Gamma\left(A_{3v}-1\right)}{8\Omega_{v}}\sum_{i=0}^{n-r} \sum_{j=0}^{i} \left(\begin{matrix} n-r \\ \text{ }i \end{matrix}\right)\left(\begin{matrix} i \\ \text{j} \end{matrix}\right)\text{B}_{1v}^{-A_{1v}}\text{B}_{2v}^{-A_{2v}}\text{B}_{3v}^{-\left(A_{3v}-1\right)}B\left(A_{0v},C_{0v}\right)B\left(B_{0v},A_{0v}+C_{0v}\right)\right\}^{\frac{1}{2}}$$

$$\hat{p}_{1v}=\left\{\frac{\Gamma\left(A_{1v}\right)\Gamma\left(A_{2v}\right)\Gamma\left(A_{3v}\right)}{8\Omega_{v}}\sum_{i=0}^{n-r} \sum_{j=0}^{i} \left(\begin{matrix} n-r \\ \text{ }i \end{matrix}\right)\left(\begin{matrix} i \\ \text{j} \end{matrix}\right)\text{B}_{1v}^{-A_{1v}}\text{B}_{2v}^{-A_{2v}}\text{B}_{3v}^{-A_{3v}}B\left(B_{0v},C_{0v}\right)B\left(A_{0v}+2,B_{0v}+C_{0v}\right)\right\}^{\frac{1}{2}}$$

$$\hat{p}_{2v}=\left\{\frac{\Gamma\left(A_{1v}\right)\Gamma\left(A_{2v}\right)\Gamma\left(A_{3v}\right)}{8\Omega_{v}}\sum_{i=0}^{n-r} \sum_{j=0}^{i} \left(\begin{matrix} n-r \\ \text{ }i \end{matrix}\right)\left(\begin{matrix} i \\ \text{j} \end{matrix}\right)\text{B}_{1v}^{-A_{1v}}\text{B}_{2v}^{-A_{2v}}\text{B}_{3v}^{-A_{3v}}B\left(A_{0v},C_{0v}\right)B\left(B_{0v}+2,A_{0v}+C_{0v}\right)\right\}^{\frac{1}{2}}$$

$$\begin{matrix} \rho\left(\hat{\lambda}_{3v}\right)=2\left\{\frac{\Gamma\left(A_{1v}\right)\Gamma\left(A_{2v}\right)\Gamma\left(A_{3v}-1\right)}{8\Omega_{v}}\sum_{i=0}^{n-r} \sum_{j=0}^{i} \left(\begin{matrix} n-r \\ \text{ }i \end{matrix}\right)\left(\begin{matrix} i \\ \text{j} \end{matrix}\right)\text{B}_{1v}^{-A_{1v}}\text{B}_{2v}^{-A_{2v}}\text{B}_{3v}^{-\left(A_{3v}-1\right)}B\left(A_{0v},C_{0v}\right)B\left(B_{0v},A_{0v}+C_{0v}\right)\right\}^{\frac{1}{2}} \\ \text{ }-2\left\{\frac{\Gamma\left(A_{1v}\right)\Gamma\left(A_{2v}\right)\Gamma\left(A_{3v}-0.5\right)}{8\Omega_{v}}\sum_{i=0}^{n-r} \sum_{j=0}^{i} \left(\begin{matrix} n-r \\ \text{ }i \end{matrix}\right)\left(\begin{matrix} i \\ \text{j} \end{matrix}\right)\text{B}_{1v}^{-A_{1v}}\text{B}_{2v}^{-A_{2v}}\text{B}_{3v}^{-\left(A_{3v}-0.5\right)}B\left(A_{0v},C_{0v}\right)B\left(B_{0v},A_{0v}+C_{0v}\right)\right\} \end{matrix}$$

$$\begin{matrix} \rho\left(\hat{p}_{1v}\right)=2\left\{\frac{\Gamma\left(A_{1v}\right)\Gamma\left(A_{2v}\right)\Gamma\left(A_{3v}\right)}{8\Omega_{v}}\sum_{i=0}^{n-r} \sum_{j=0}^{i} \left(\begin{matrix} n-r \\ \text{ }i \end{matrix}\right)\left(\begin{matrix} i \\ \text{j} \end{matrix}\right)\text{B}_{1v}^{-A_{1v}}\text{B}_{2v}^{-A_{2v}}\text{B}_{3v}^{-A_{3v}}B\left(B_{0v},C_{0v}\right)B\left(A_{0v}+2,B_{0v}+C_{0v}\right)\right\}^{\frac{1}{2}} \\ \text{ }-2\left\{\frac{\Gamma\left(A_{1v}\right)\Gamma\left(A_{2v}\right)\Gamma\left(A_{3v}\right)}{8\Omega_{v}}\sum_{i=0}^{n-r} \sum_{j=0}^{i} \left(\begin{matrix} n-r \\ \text{ }i \end{matrix}\right)\left(\begin{matrix} i \\ \text{j} \end{matrix}\right)\text{B}_{1v}^{-A_{1v}}\text{B}_{2v}^{-A_{2v}}\text{B}_{3v}^{-A_{3v}}B\left(B_{0v},C_{0v}\right)B\left(A_{0v}+1,B_{0v}+C_{0v}\right)\right\} \end{matrix}$$

$$\begin{matrix} \rho\left(\hat{p}_{2v}\right)=2\left\{\frac{\Gamma\left(A_{1v}\right)\Gamma\left(A_{2v}\right)\Gamma\left(A_{3v}\right)}{8\Omega_{v}}\sum_{i=0}^{n-r} \sum_{j=0}^{i} \left(\begin{matrix} n-r \\ \text{ }i \end{matrix}\right)\left(\begin{matrix} i \\ \text{j} \end{matrix}\right)\text{B}_{1v}^{-A_{1v}}\text{B}_{2v}^{-A_{2v}}\text{B}_{3v}^{-A_{3v}}B\left(A_{0v},C_{0v}\right)B\left(B_{0v}+2,A_{0v}+C_{0v}\right)\right\}^{\frac{1}{2}} \\ \text{ }-2\left\{\frac{\Gamma\left(A_{1v}\right)\Gamma\left(A_{2v}\right)\Gamma\left(A_{3v}\right)}{8\Omega_{v}}\sum_{i=0}^{n-r} \sum_{j=0}^{i} \left(\begin{matrix} n-r \\ \text{ }i \end{matrix}\right)\left(\begin{matrix} i \\ \text{j} \end{matrix}\right)\text{B}_{1v}^{-A_{1v}}\text{B}_{2v}^{-A_{2v}}\text{B}_{3v}^{-A_{3v}}B\left(A_{0v},C_{0v}\right)B\left(B_{0v}+1,A_{0v}+C_{0v}\right)\right\} \end{matrix}$$

**Bayes estimators and posterior risks using the UP, the JP, the ICP and the SRIGP under DLF**

$$\hat{\lambda}_{1v}=\frac{\Gamma\left(A_{1v}-1\right)\Gamma\left(A_{2v}\right)\Gamma\left(A_{3v}\right)\sum_{i=0}^{n-r} \sum_{j=0}^{i} \left(\begin{matrix} n-r \\ \text{ }i \end{matrix}\right)\left(\begin{matrix} i \\ \text{j} \end{matrix}\right)\text{B}_{1v}^{-\left(A_{1v}-1\right)}\text{B}_{2v}^{-A_{2v}}\text{B}_{3v}^{-A_{3v}}B\left(A_{0v},C_{0v}\right)B\left(B_{0v},A_{0v}+C_{0v}\right)}{\Gamma\left(A_{1v}-0.5\right)\Gamma\left(A_{2v}\right)\Gamma\left(A_{3v}\right)\sum_{i=0}^{n-r} \sum_{j=0}^{i} \left(\begin{matrix} n-r \\ \text{ }i \end{matrix}\right)\left(\begin{matrix} i \\ \text{j} \end{matrix}\right)\text{B}_{1v}^{-\left(A_{1v}-\frac{1}{2}\right)}\text{B}_{2v}^{-A_{2v}}\text{B}_{3v}^{-A_{3v}}B\left(A_{0v},C_{0v}\right)B\left(B_{0v},A_{0v}+C_{0v}\right)}$$

$$\hat{\lambda}_{2v}=\frac{\Gamma\left(A_{1v}\right)\Gamma\left(A_{2v}-1\right)\Gamma\left(A_{3v}\right)\sum_{i=0}^{n-r} \sum_{j=0}^{i} \left(\begin{matrix} n-r \\ \text{ }i \end{matrix}\right)\left(\begin{matrix} i \\ \text{j} \end{matrix}\right)\text{B}_{1v}^{-A_{1v}}\text{B}_{2v}^{-\left(A_{2v}-1\right)}\text{B}_{3v}^{-A_{3v}}B\left(A_{0v},C_{0v}\right)B\left(B_{0v},A_{0v}+C_{0v}\right)}{\Gamma\left(A_{1v}\right)\Gamma\left(A_{2v}-0.5\right)\Gamma\left(A_{3v}\right)\sum_{i=0}^{n-r} \sum_{j=0}^{i} \left(\begin{matrix} n-r \\ \text{ }i \end{matrix}\right)\left(\begin{matrix} i \\ \text{j} \end{matrix}\right)\text{B}_{1v}^{-A_{1v}}\text{B}_{2v}^{-\left(A_{2v}-\frac{1}{2}\right)}\text{B}_{3v}^{-A_{3v}}B\left(A_{0v},C_{0v}\right)B\left(B_{0v},A_{0v}+C_{0v}\right)}$$

$$\hat{\lambda}_{3v}=\frac{\Gamma\left(A_{1v}\right)\Gamma\left(A_{2v}\right)\Gamma\left(A_{3v}-1\right)\sum_{i=0}^{n-r} \sum_{j=0}^{i} \left(\begin{matrix} n-r \\ \text{ }i \end{matrix}\right)\left(\begin{matrix} i \\ \text{j} \end{matrix}\right)\text{B}_{1v}^{-A_{1v}}\text{B}_{2v}^{-A_{2v}}\text{B}_{3v}^{-\left(A_{3v}-1\right)}B\left(A_{0v},C_{0v}\right)B\left(B_{0v},A_{0v}+C_{0v}\right)}{\Gamma\left(A_{1v}\right)\Gamma\left(A_{2v}\right)\Gamma\left(A_{3v}-0.5\right)\sum_{i=0}^{n-r} \sum_{j=0}^{i} \left(\begin{matrix} n-r \\ \text{ }i \end{matrix}\right)\left(\begin{matrix} i \\ \text{j} \end{matrix}\right)\text{B}_{1v}^{-A_{1v}}\text{B}_{2v}^{-A_{2v}}\text{B}_{3v}^{-\left(A_{3v}-\frac{1}{2}\right)}B\left(A_{0v},C_{0v}\right)B\left(B_{0v},A_{0v}+C_{0v}\right)}$$

$$\hat{p}_{1v}=\frac{\Gamma\left(A_{1v}\right)\Gamma\left(A_{2v}\right)\Gamma\left(A_{3v}\right)\sum_{i=0}^{n-r} \sum_{j=0}^{i} \left(\begin{matrix} n-r \\ \text{ }i \end{matrix}\right)\left(\begin{matrix} i \\ \text{j} \end{matrix}\right)\text{B}_{1v}^{-A_{1v}}\text{B}_{2v}^{-A_{2v}}\text{B}_{3v}^{-A_{3v}}B\left(B_{0v},C_{0v}\right)B\left(A_{0v}+2,B_{0v}+C_{0v}\right)}{\Gamma\left(A_{1v}\right)\Gamma\left(A_{2v}\right)\Gamma\left(A_{3v}\right)\sum_{i=0}^{n-r} \sum_{j=0}^{i} \left(\begin{matrix} n-r \\ \text{ }i \end{matrix}\right)\left(\begin{matrix} i \\ \text{j} \end{matrix}\right)\text{B}_{1v}^{-A_{1v}}\text{B}_{2v}^{-A_{2v}}\text{B}_{3v}^{-A_{3v}}B\left(B_{0v},C_{0v}\right)B\left(A_{0v}+1,B_{0v}+C_{0v}\right)}$$

$$\hat{p}_{2v}=\frac{\Gamma\left(A_{1v}\right)\Gamma\left(A_{2v}\right)\Gamma\left(A_{3v}\right)\sum_{i=0}^{n-r} \sum_{j=0}^{i} \left(\begin{matrix} n-r \\ \text{ }i \end{matrix}\right)\left(\begin{matrix} i \\ \text{j} \end{matrix}\right)\text{B}_{1v}^{-A_{1v}}\text{B}_{2v}^{-A_{2v}}\text{B}_{3v}^{-A_{3v}}B\left(A_{0v},C_{0v}\right)B\left(B_{0v}+2,A_{0v}+C_{0v}\right)}{\Gamma\left(A_{1v}\right)\Gamma\left(A_{2v}\right)\Gamma\left(A_{3v}\right)\sum_{i=0}^{n-r} \sum_{j=0}^{i} \left(\begin{matrix} n-r \\ \text{ }i \end{matrix}\right)\left(\begin{matrix} i \\ \text{j} \end{matrix}\right)\text{B}_{1v}^{-A_{1v}}\text{B}_{2v}^{-A_{2v}}\text{B}_{3v}^{-A_{3v}}B\left(A_{0v},C_{0v}\right)B\left(B_{0v}+1,A_{0v}+C_{0v}\right)}$$

$$\rho\left(\hat{\lambda}_{3v}\right)=1-\frac{\left\{\Gamma\left(A_{1v}\right)\Gamma\left(A_{2v}\right)\Gamma\left(A_{3v}-0.5\right)\sum_{i=0}^{n-r} \sum_{j=0}^{i} \left(\begin{matrix} n-r \\ \text{ }i \end{matrix}\right)\left(\begin{matrix} i \\ \text{j} \end{matrix}\right)\text{B}_{1v}^{-A_{1v}}\text{B}_{2v}^{-A_{2v}}\text{B}_{3v}^{-\left(A_{3v}-0.5\right)}B\left(A_{0v},C_{0v}\right)B\left(B_{0v},A_{0v}+C_{0v}\right)\right\}^{2}}{8\Omega_{v}\Gamma\left(A_{1v}\right)\Gamma\left(A_{2v}\right)\Gamma\left(A_{3v}-1\right)\sum_{i=0}^{n-r} \sum_{j=0}^{i} \left(\begin{matrix} n-r \\ \text{ }i \end{matrix}\right)\left(\begin{matrix} i \\ \text{j} \end{matrix}\right)\text{B}_{1v}^{-A_{1v}}\text{B}_{2v}^{-A_{2v}}\text{B}_{3v}^{-\left(A_{3v}-1\right)}B\left(A_{0v},C_{0v}\right)B\left(B_{0v},A_{0v}+C_{0v}\right)}$$

$$\rho\left(\hat{p}_{1v}\right)=1-\frac{\left\{\Gamma\left(A_{1v}\right)\Gamma\left(A_{2v}\right)\Gamma\left(A_{3v}\right)\sum_{i=0}^{n-r} \sum_{j=0}^{i} \left(\begin{matrix} n-r \\ \text{ }i \end{matrix}\right)\left(\begin{matrix} i \\ \text{j} \end{matrix}\right)\text{B}_{1v}^{-A_{1v}}\text{B}_{2v}^{-A_{2v}}\text{B}_{3v}^{-A_{3v}}B\left(B_{0v},C_{0v}\right)B\left(A_{0v}+1,B_{0v}+C_{02}\right)\right\}^{2}}{8\Omega_{v}\Gamma\left(A_{1v}\right)\Gamma\left(A_{2v}\right)\Gamma\left(A_{3v}\right)\sum_{i=0}^{n-r} \sum_{j=0}^{i} \left(\begin{matrix} n-r \\ \text{ }i \end{matrix}\right)\left(\begin{matrix} i \\ \text{j} \end{matrix}\right)\text{B}_{1v}^{-A_{1v}}\text{B}_{2v}^{-A_{2v}}\text{B}_{3v}^{-A_{3v}}B\left(B_{0v},C_{0v}\right)B\left(A_{0v}+2,B_{0v}+C_{02}\right)}$$

$$\rho\left(\hat{p}_{2v}\right)=1-\frac{\left\{\Gamma\left(A_{1v}\right)\Gamma\left(A_{2v}\right)\Gamma\left(A_{3v}\right)\sum_{i=0}^{n-r} \sum_{j=0}^{i} \left(\begin{matrix} n-r \\ \text{ }i \end{matrix}\right)\left(\begin{matrix} i \\ \text{j} \end{matrix}\right)\text{B}_{1v}^{-A_{1v}}\text{B}_{2v}^{-A_{2v}}\text{B}_{3v}^{-A_{3v}}B\left(A_{0v},C_{0v}\right)B\left(B_{0v}+1,A_{0v}+C_{0v}\right)\right\}^{2}}{8\Omega_{v}\Gamma\left(A_{1v}\right)\Gamma\left(A_{2v}\right)\Gamma\left(A_{3v}\right)\sum_{i=0}^{n-r} \sum_{j=0}^{i} \left(\begin{matrix} n-r \\ \text{ }i \end{matrix}\right)\left(\begin{matrix} i \\ \text{j} \end{matrix}\right)\text{B}_{1v}^{-A_{1v}}\text{B}_{2v}^{-A_{2v}}\text{B}_{3v}^{-A_{3v}}B\left(A_{0v},C_{0v}\right)B\left(B_{0v}+2,A_{0v}+C_{0v}\right)}$$
