# Supplementary material for: A 3-Component Mixture of Rayleigh Distributions: Properties and Estimation in Bayesian Framework
Source: PLoS One. 2015 May 20;10(5):e0126183. doi: 10.1371/journal.pone.0126183 (PMC4439070; doi:10.1371/journal.pone.0126183)
Supplement: S2 File — (DOC) [file pone.0126183.s002.doc]

File S2: Limiting expressions for the Bayes estimators.

Table A: Limiting expressions for the Bayes estimators as $t\to\infty$using the UP and the JP under SELF.

| Parameters | Bayes Estimators | |
| --- | --- | --- |
|  | UP | JP |
|  | $\frac{\left(\frac{1}{2}\sum_{k=1}^{n_{1}} y_{1k}^{2}\right)^{1/2}\Gamma\left(n_{1}-1\right)}{\Gamma\left(n_{1}-\frac{1}{2}\right)}$ | $\frac{\left(\frac{1}{2}\sum_{k=1}^{n_{1}} y_{1k}^{2}\right)^{1/2}\Gamma\left(n_{1}-\frac{1}{2}\right)}{\Gamma\left(n_{1}\right)}$ |
|  | $\frac{\left(\frac{1}{2}\sum_{k=1}^{n_{2}} y_{2k}^{2}\right)^{1/2}\Gamma\left(n_{2}-1\right)}{\Gamma\left(n_{2}-\frac{1}{2}\right)}$ | $\frac{\left(\frac{1}{2}\sum_{k=1}^{n_{2}} y_{2k}^{2}\right)^{1/2}\Gamma\left(n_{2}-\frac{1}{2}\right)}{\Gamma\left(n_{2}\right)}$ |
|  | $\frac{\left(\frac{1}{2}\sum_{k=1}^{n_{3}} y_{3k}^{2}\right)^{1/2}\Gamma\left(n_{3}-1\right)}{\Gamma\left(n_{3}-\frac{1}{2}\right)}$ | $\frac{\left(\frac{1}{2}\sum_{k=1}^{n_{3}} y_{3k}^{2}\right)^{1/2}\Gamma\left(n_{3}-\frac{1}{2}\right)}{\Gamma\left(n_{3}\right)}$ |
|  | $\frac{n_{1}+1}{n+3}$ | $\frac{n_{1}+1}{n+3}$ |
|  | $\frac{n_{2}+1}{n+3}$ | $\frac{n_{2}+1}{n+3}$ |

Table B: Limiting expressions for the Bayes estimators as $t\to\infty$using the ICP and the SRIGP under SELF.

| Parameters | Bayes Estimators | |
| --- | --- | --- |
|  | ICP | SRIGP |
|  | $\frac{\left(\frac{1}{2}\sum_{k=1}^{n_{1}} y_{1k}^{2}+\frac{b_{1}}{2}\right)^{1/2}\Gamma\left(n_{1}+\frac{a_{1}}{2}-\frac{1}{2}\right)}{\Gamma\left(n_{1}+\frac{a_{1}}{2}\right)}$ | $\frac{\left(\frac{1}{2}\sum_{k=1}^{n_{1}} y_{1k}^{2}+b_{1}\right)^{1/2}\Gamma\left(n_{1}+a_{1}-\frac{1}{2}\right)}{\Gamma\left(n_{1}+a_{1}\right)}$ |
|  | $\frac{\left(\frac{1}{2}\sum_{k=1}^{n_{2}} y_{2k}^{2}+\frac{b_{2}}{2}\right)^{1/2}\Gamma\left(n_{2}+\frac{a_{2}}{2}-\frac{1}{2}\right)}{\Gamma\left(n_{2}+\frac{a_{2}}{2}\right)}$ | $\frac{\left(\frac{1}{2}\sum_{k=1}^{n_{2}} y_{2k}^{2}+b_{2}\right)^{1/2}\Gamma\left(n_{2}+a_{2}-\frac{1}{2}\right)}{\Gamma\left(n_{2}+a_{2}\right)}$ |
|  | $\frac{\left(\frac{1}{2}\sum_{k=1}^{n_{3}} y_{3k}^{2}+\frac{b_{3}}{2}\right)^{1/2}\Gamma\left(n_{3}+\frac{a_{3}}{2}-\frac{1}{2}\right)}{\Gamma\left(n_{3}+\frac{a_{3}}{2}\right)}$ | $\frac{\left(\frac{1}{2}\sum_{k=1}^{n_{3}} y_{3k}^{2}+b_{3}\right)^{1/2}\Gamma\left(n_{3}+a_{3}-\frac{1}{2}\right)}{\Gamma\left(n_{3}+a_{3}\right)}$ |
|  | $\frac{n_{1}+a}{n+a+b+c}$ | $\frac{n_{1}+a}{n+a+b+c}$ |
|  | $\frac{n_{2}+b}{n+a+b+c}$ | $\frac{n_{2}+b}{n+a+b+c}$ |

Table C: Limiting expressions for the posterior risks as $t\to\infty$using the UP and the JP under SELF.

| Parameters | Posterior Risks | |
| --- | --- | --- |
|  | UP | JP |
|  | $\left(\frac{1}{2}\sum_{k=1}^{n_{1}} y_{1k}^{2}\right)\left[\frac{1}{\left(n_{1}-\frac{3}{2}\right)}-\left\{\frac{\Gamma\left(n_{1}-1\right)}{\Gamma\left(n_{1}-\frac{1}{2}\right)}\right\}^{2}\right]$ | $\left(\frac{1}{2}\sum_{k=1}^{n_{1}} y_{1k}^{2}\right)\left[\frac{1}{\left(n_{1}-1\right)}-\left\{\frac{\Gamma\left(n_{1}-\frac{1}{2}\right)}{\Gamma\left(n_{1}\right)}\right\}^{2}\right]$ |
|  | $\left(\frac{1}{2}\sum_{k=1}^{n_{2}} y_{2k}^{2}\right)\left[\frac{1}{\left(n_{2}-\frac{3}{2}\right)}-\left\{\frac{\Gamma\left(n_{2}-1\right)}{\Gamma\left(n_{2}-\frac{1}{2}\right)}\right\}^{2}\right]$ | $\left(\frac{1}{2}\sum_{k=1}^{n_{2}} y_{2k}^{2}\right)\left[\frac{1}{\left(n_{2}-1\right)}-\left\{\frac{\Gamma\left(n_{2}-\frac{1}{2}\right)}{\Gamma\left(n_{2}\right)}\right\}^{2}\right]$ |
|  | $\left(\frac{1}{2}\sum_{k=1}^{n_{3}} y_{3k}^{2}\right)\left[\frac{1}{\left(n_{3}-\frac{3}{2}\right)}-\left\{\frac{\Gamma\left(n_{3}-1\right)}{\Gamma\left(n_{3}-\frac{1}{2}\right)}\right\}^{2}\right]$ | $\left(\frac{1}{2}\sum_{k=1}^{n_{3}} y_{3k}^{2}\right)\left[\frac{1}{\left(n_{3}-1\right)}-\left\{\frac{\Gamma\left(n_{3}-\frac{1}{2}\right)}{\Gamma\left(n_{3}\right)}\right\}^{2}\right]$ |
|  | $\frac{\left(n_{1}+1\right)\left(n_{2}+n_{3}+2\right)}{\left(n+3\right)^{2}\left(n+4\right)}$ | $\frac{\left(n_{1}+1\right)\left(n_{2}+n_{3}+2\right)}{\left(n+3\right)^{2}\left(n+4\right)}$ |
|  | $\frac{\left(n_{2}+1\right)\left(n_{1}+n_{3}+2\right)}{\left(n+3\right)^{2}\left(n+4\right)}$ | $\frac{\left(n_{2}+1\right)\left(n_{1}+n_{3}+2\right)}{\left(n+3\right)^{2}\left(n+4\right)}$ |

Table D: Limiting expressions for the posterior risks as $t\to\infty$using the ICP and the SRIGP under SELF.

| Param-eters | Posterior Risks | |
| --- | --- | --- |
|  | ICP | SRIGP |
|  | $\left(\frac{1}{2}\sum_{k=1}^{n_{1}} y_{1k}^{2}+\frac{b_{1}}{2}\right)\left[\frac{1}{\left(n_{1}+\frac{a_{1}}{2}-1\right)}-\left\{\frac{\Gamma\left(n_{1}+\frac{a_{1}}{2}-\frac{1}{2}\right)}{\Gamma\left(n_{1}+\frac{a_{1}}{2}\right)}\right\}^{2}\right]$ | $\left(\frac{1}{2}\sum_{k=1}^{n_{1}} y_{1k}^{2}+b_{1}\right)\left[\frac{1}{\left(n_{1}+a_{1}-1\right)}-\left\{\frac{\Gamma\left(n_{1}+a_{1}-\frac{1}{2}\right)}{\Gamma\left(n_{1}+a_{1}\right)}\right\}^{2}\right]$ |
|  | $\left(\frac{1}{2}\sum_{k=1}^{n_{2}} y_{2k}^{2}+\frac{b_{2}}{2}\right)\left[\frac{1}{\left(n_{2}+\frac{a_{2}}{2}-1\right)}-\left\{\frac{\Gamma\left(n_{2}+\frac{a_{2}}{2}-\frac{1}{2}\right)}{\Gamma\left(n_{2}+\frac{a_{2}}{2}\right)}\right\}^{2}\right]$ | $\left(\frac{1}{2}\sum_{k=1}^{n_{2}} y_{2k}^{2}+b_{2}\right)\left[\frac{1}{\left(n_{2}+a_{2}-1\right)}-\left\{\frac{\Gamma\left(n_{2}+a_{2}-\frac{1}{2}\right)}{\Gamma\left(n_{2}+a_{2}\right)}\right\}^{2}\right]$ |
|  | $\left(\frac{1}{2}\sum_{k=1}^{n_{3}} y_{3k}^{2}+\frac{b_{3}}{2}\right)\left[\frac{1}{\left(n_{3}+\frac{a_{3}}{2}-1\right)}-\left\{\frac{\Gamma\left(n_{3}+\frac{a_{3}}{2}-\frac{1}{2}\right)}{\Gamma\left(n_{3}+\frac{a_{3}}{2}\right)}\right\}^{2}\right]$ | $\left(\frac{1}{2}\sum_{k=1}^{n_{3}} y_{3k}^{2}+b_{3}\right)\left[\frac{1}{\left(n_{3}+a_{3}-1\right)}-\left\{\frac{\Gamma\left(n_{3}+a_{3}-\frac{1}{2}\right)}{\Gamma\left(n_{3}+a_{3}\right)}\right\}^{2}\right]$ |
|  | $\frac{\left(n_{1}+a\right)\left(n_{2}+n_{3}+b+c\right)}{\left(n+a+b+c\right)^{2}\left(n+a+b+c+1\right)}$ | $\frac{\left(n_{1}+a\right)\left(n_{2}+n_{3}+b+c\right)}{\left(n+a+b+c\right)^{2}\left(n+a+b+c+1\right)}$ |
|  | $\frac{\left(n_{2}+b\right)\left(n_{1}+n_{3}+a+c\right)}{\left(n+a+b+c\right)^{2}\left(n+a+b+c+1\right)}$ | $\frac{\left(n_{2}+b\right)\left(n_{1}+n_{3}+a+c\right)}{\left(n+a+b+c\right)^{2}\left(n+a+b+c+1\right)}$ |
