# Supplementary material for: A 3-Component Mixture of Rayleigh Distributions: Properties and Estimation in Bayesian Framework
Source: PLoS One. 2015 May 20;10(5):e0126183. doi: 10.1371/journal.pone.0126183 (PMC4439070; doi:10.1371/journal.pone.0126183)
Supplement: S1 Table — (DOCX) [file pone.0126183.s003.docx]

Table S1: Limiting expressions for the Bayes estimators as $t\to\infty$ using the UP and the JP under SELF.

| Parameters | Bayes Estimators | |
| --- | --- | --- |
|  | UP | JP |
|  | $\frac{\left(\frac{1}{2}\sum_{k=1}^{n_{1}} y_{1k}^{2}\right)^{1/2}\Gamma\left(n_{1}-1\right)}{\Gamma\left(n_{1}-\frac{1}{2}\right)}$ | $\frac{\left(\frac{1}{2}\sum_{k=1}^{n_{1}} y_{1k}^{2}\right)^{1/2}\Gamma\left(n_{1}-\frac{1}{2}\right)}{\Gamma\left(n_{1}\right)}$ |
|  | $\frac{\left(\frac{1}{2}\sum_{k=1}^{n_{2}} y_{2k}^{2}\right)^{1/2}\Gamma\left(n_{2}-1\right)}{\Gamma\left(n_{2}-\frac{1}{2}\right)}$ | $\frac{\left(\frac{1}{2}\sum_{k=1}^{n_{2}} y_{2k}^{2}\right)^{1/2}\Gamma\left(n_{2}-\frac{1}{2}\right)}{\Gamma\left(n_{2}\right)}$ |
|  | $\frac{\left(\frac{1}{2}\sum_{k=1}^{n_{3}} y_{3k}^{2}\right)^{1/2}\Gamma\left(n_{3}-1\right)}{\Gamma\left(n_{3}-\frac{1}{2}\right)}$ | $\frac{\left(\frac{1}{2}\sum_{k=1}^{n_{3}} y_{3k}^{2}\right)^{1/2}\Gamma\left(n_{3}-\frac{1}{2}\right)}{\Gamma\left(n_{3}\right)}$ |
|  | $\frac{n_{1}+1}{n+3}$ | $\frac{n_{1}+1}{n+3}$ |
|  | $\frac{n_{2}+1}{n+3}$ | $\frac{n_{2}+1}{n+3}$ |
