# Supplementary material for: A 3-Component Mixture of Rayleigh Distributions: Properties and Estimation in Bayesian Framework
Source: PLoS One. 2015 May 20;10(5):e0126183. doi: 10.1371/journal.pone.0126183 (PMC4439070; doi:10.1371/journal.pone.0126183)
Supplement: S2 Table — (DOCX) [file pone.0126183.s004.docx]

Table S2: Limiting expressions for the Bayes estimators as $t\to\infty$ using the ICP and the SRIGP under SELF.

| Parameters | Bayes Estimators | |
| --- | --- | --- |
|  | ICP | SRIGP |
|  | $\frac{\left(\frac{1}{2}\sum_{k=1}^{n_{1}} y_{1k}^{2}+\frac{b_{1}}{2}\right)^{1/2}\Gamma\left(n_{1}+\frac{a_{1}}{2}-\frac{1}{2}\right)}{\Gamma\left(n_{1}+\frac{a_{1}}{2}\right)}$ | $\frac{\left(\frac{1}{2}\sum_{k=1}^{n_{1}} y_{1k}^{2}+b_{1}\right)^{1/2}\Gamma\left(n_{1}+a_{1}-\frac{1}{2}\right)}{\Gamma\left(n_{1}+a_{1}\right)}$ |
|  | $\frac{\left(\frac{1}{2}\sum_{k=1}^{n_{2}} y_{2k}^{2}+\frac{b_{2}}{2}\right)^{1/2}\Gamma\left(n_{2}+\frac{a_{2}}{2}-\frac{1}{2}\right)}{\Gamma\left(n_{2}+\frac{a_{2}}{2}\right)}$ | $\frac{\left(\frac{1}{2}\sum_{k=1}^{n_{2}} y_{2k}^{2}+b_{2}\right)^{1/2}\Gamma\left(n_{2}+a_{2}-\frac{1}{2}\right)}{\Gamma\left(n_{2}+a_{2}\right)}$ |
|  | $\frac{\left(\frac{1}{2}\sum_{k=1}^{n_{3}} y_{3k}^{2}+\frac{b_{3}}{2}\right)^{1/2}\Gamma\left(n_{3}+\frac{a_{3}}{2}-\frac{1}{2}\right)}{\Gamma\left(n_{3}+\frac{a_{3}}{2}\right)}$ | $\frac{\left(\frac{1}{2}\sum_{k=1}^{n_{3}} y_{3k}^{2}+b_{3}\right)^{1/2}\Gamma\left(n_{3}+a_{3}-\frac{1}{2}\right)}{\Gamma\left(n_{3}+a_{3}\right)}$ |
|  | $\frac{n_{1}+a}{n+a+b+c}$ | $\frac{n_{1}+a}{n+a+b+c}$ |
|  | $\frac{n_{2}+b}{n+a+b+c}$ | $\frac{n_{2}+b}{n+a+b+c}$ |
