# Supplementary material for: A 3-Component Mixture of Rayleigh Distributions: Properties and Estimation in Bayesian Framework
Source: PLoS One. 2015 May 20;10(5):e0126183. doi: 10.1371/journal.pone.0126183 (PMC4439070; doi:10.1371/journal.pone.0126183)
Supplement: S3 Table — (DOCX) [file pone.0126183.s005.docx]

Table S3: Limiting expressions for the posterior risks as $t\to\infty$ using the UP and the JP under SELF.

| Parameters | Posterior Risks | |
| --- | --- | --- |
|  | UP | JP |
|  | $\left(\frac{1}{2}\sum_{k=1}^{n_{1}} y_{1k}^{2}\right)\left[\frac{1}{\left(n_{1}-\frac{3}{2}\right)}-\left\{\frac{\Gamma\left(n_{1}-1\right)}{\Gamma\left(n_{1}-\frac{1}{2}\right)}\right\}^{2}\right]$ | $\left(\frac{1}{2}\sum_{k=1}^{n_{1}} y_{1k}^{2}\right)\left[\frac{1}{\left(n_{1}-1\right)}-\left\{\frac{\Gamma\left(n_{1}-\frac{1}{2}\right)}{\Gamma\left(n_{1}\right)}\right\}^{2}\right]$ |
|  | $\left(\frac{1}{2}\sum_{k=1}^{n_{2}} y_{2k}^{2}\right)\left[\frac{1}{\left(n_{2}-\frac{3}{2}\right)}-\left\{\frac{\Gamma\left(n_{2}-1\right)}{\Gamma\left(n_{2}-\frac{1}{2}\right)}\right\}^{2}\right]$ | $\left(\frac{1}{2}\sum_{k=1}^{n_{2}} y_{2k}^{2}\right)\left[\frac{1}{\left(n_{2}-1\right)}-\left\{\frac{\Gamma\left(n_{2}-\frac{1}{2}\right)}{\Gamma\left(n_{2}\right)}\right\}^{2}\right]$ |
|  | $\left(\frac{1}{2}\sum_{k=1}^{n_{3}} y_{3k}^{2}\right)\left[\frac{1}{\left(n_{3}-\frac{3}{2}\right)}-\left\{\frac{\Gamma\left(n_{3}-1\right)}{\Gamma\left(n_{3}-\frac{1}{2}\right)}\right\}^{2}\right]$ | $\left(\frac{1}{2}\sum_{k=1}^{n_{3}} y_{3k}^{2}\right)\left[\frac{1}{\left(n_{3}-1\right)}-\left\{\frac{\Gamma\left(n_{3}-\frac{1}{2}\right)}{\Gamma\left(n_{3}\right)}\right\}^{2}\right]$ |
|  | $\frac{\left(n_{1}+1\right)\left(n_{2}+n_{3}+2\right)}{\left(n+3\right)^{2}\left(n+4\right)}$ | $\frac{\left(n_{1}+1\right)\left(n_{2}+n_{3}+2\right)}{\left(n+3\right)^{2}\left(n+4\right)}$ |
|  | $\frac{\left(n_{2}+1\right)\left(n_{1}+n_{3}+2\right)}{\left(n+3\right)^{2}\left(n+4\right)}$ | $\frac{\left(n_{2}+1\right)\left(n_{1}+n_{3}+2\right)}{\left(n+3\right)^{2}\left(n+4\right)}$ |
