# Supplementary material for: A 3-Component Mixture of Rayleigh Distributions: Properties and Estimation in Bayesian Framework
Source: PLoS One. 2015 May 20;10(5):e0126183. doi: 10.1371/journal.pone.0126183 (PMC4439070; doi:10.1371/journal.pone.0126183)
Supplement: S4 Table — (DOCX) [file pone.0126183.s006.docx]

Table S4: Limiting expressions for the posterior risks as $t\to\infty$ using the ICP and the SRIGP under SELF.

| Param-eters | Posterior Risks | |
| --- | --- | --- |
|  | ICP | SRIGP |
|  | $\left(\frac{1}{2}\sum_{k=1}^{n_{1}} y_{1k}^{2}+\frac{b_{1}}{2}\right)\left[\frac{1}{\left(n_{1}+\frac{a_{1}}{2}-1\right)}-\left\{\frac{\Gamma\left(n_{1}+\frac{a_{1}}{2}-\frac{1}{2}\right)}{\Gamma\left(n_{1}+\frac{a_{1}}{2}\right)}\right\}^{2}\right]$ | $\left(\frac{1}{2}\sum_{k=1}^{n_{1}} y_{1k}^{2}+b_{1}\right)\left[\frac{1}{\left(n_{1}+a_{1}-1\right)}-\left\{\frac{\Gamma\left(n_{1}+a_{1}-\frac{1}{2}\right)}{\Gamma\left(n_{1}+a_{1}\right)}\right\}^{2}\right]$ |
|  | $\left(\frac{1}{2}\sum_{k=1}^{n_{2}} y_{2k}^{2}+\frac{b_{2}}{2}\right)\left[\frac{1}{\left(n_{2}+\frac{a_{2}}{2}-1\right)}-\left\{\frac{\Gamma\left(n_{2}+\frac{a_{2}}{2}-\frac{1}{2}\right)}{\Gamma\left(n_{2}+\frac{a_{2}}{2}\right)}\right\}^{2}\right]$ | $\left(\frac{1}{2}\sum_{k=1}^{n_{2}} y_{2k}^{2}+b_{2}\right)\left[\frac{1}{\left(n_{2}+a_{2}-1\right)}-\left\{\frac{\Gamma\left(n_{2}+a_{2}-\frac{1}{2}\right)}{\Gamma\left(n_{2}+a_{2}\right)}\right\}^{2}\right]$ |
|  | $\left(\frac{1}{2}\sum_{k=1}^{n_{3}} y_{3k}^{2}+\frac{b_{3}}{2}\right)\left[\frac{1}{\left(n_{3}+\frac{a_{3}}{2}-1\right)}-\left\{\frac{\Gamma\left(n_{3}+\frac{a_{3}}{2}-\frac{1}{2}\right)}{\Gamma\left(n_{3}+\frac{a_{3}}{2}\right)}\right\}^{2}\right]$ | $\left(\frac{1}{2}\sum_{k=1}^{n_{3}} y_{3k}^{2}+b_{3}\right)\left[\frac{1}{\left(n_{3}+a_{3}-1\right)}-\left\{\frac{\Gamma\left(n_{3}+a_{3}-\frac{1}{2}\right)}{\Gamma\left(n_{3}+a_{3}\right)}\right\}^{2}\right]$ |
|  | $\frac{\left(n_{1}+a\right)\left(n_{2}+n_{3}+b+c\right)}{\left(n+a+b+c\right)^{2}\left(n+a+b+c+1\right)}$ | $\frac{\left(n_{1}+a\right)\left(n_{2}+n_{3}+b+c\right)}{\left(n+a+b+c\right)^{2}\left(n+a+b+c+1\right)}$ |
|  | $\frac{\left(n_{2}+b\right)\left(n_{1}+n_{3}+a+c\right)}{\left(n+a+b+c\right)^{2}\left(n+a+b+c+1\right)}$ | $\frac{\left(n_{2}+b\right)\left(n_{1}+n_{3}+a+c\right)}{\left(n+a+b+c\right)^{2}\left(n+a+b+c+1\right)}$ |
