# Supplementary material for: A 3-Component Mixture of Rayleigh Distributions: Properties and Estimation in Bayesian Framework
Source: PLoS One. 2015 May 20;10(5):e0126183. doi: 10.1371/journal.pone.0126183 (PMC4439070; doi:10.1371/journal.pone.0126183)
Supplement: S5 Table — (DOCX) [file pone.0126183.s007.docx]

Table S5: The BEs and the PRs using the UP with and

|  |  | Loss Functions | | UP | | | | |
| --- | --- | --- | --- | --- | --- | --- | --- | --- |
|  |  |  |  |  |  |  |  |  |
| 25 | 50 | SELF | BE | 13.61660 | 12.92240 | 11.76780 | 0.461779 | 0.312864 |
|  |  |  | PR | **4.532860** | **7.302360** | **9.800650** | **0.006105** | **0.005259** |
|  |  | PLF | BE | 13.65180 | 13.21880 | 12.15650 | 0.468459 | 0.321551 |
|  |  |  | PR | **0.317153** | **0.535797** | **0.766400** | **0.013103** | **0.016509** |
|  |  | DLF | BE | 13.74200 | 13.44650 | 12.59640 | 0.475416 | 0.328461 |
|  |  |  | PR | **0.022405** | **0.038845** | **0.059318** | **0.027628** | **0.050744** |
|  | 100 | SELF | BE | 13.61890 | 12.65150 | 11.04510 | 0.473665 | 0.311142 |
|  |  |  | PR | **2.267110** | **3.672770** | **4.200150** | **0.003369** | **0.002903** |
|  |  | PLF | BE | 13.69180 | 12.75010 | 11.24570 | 0.478501 | 0.314372 |
|  |  |  | PR | **0.162659** | **0.281566** | **0.360224** | **0.007079** | **0.009209** |
|  |  | DLF | BE | 13.75820 | 12.87130 | 11.39920 | 0.482014 | 0.319356 |
|  |  |  | PR | **0.011877** | **0.021755** | **0.031074** | **0.014863** | **0.029082** |
|  | 200 | SELF | BE | 13.66825 | 12.38945 | 10.50893 | 0.483701 | 0.308232 |
|  |  |  | PR | **1.126665** | **1.852856** | **1.769164** | **0.001778** | **0.001526** |
|  |  | PLF | BE | 13.72018 | 12.46556 | 10.63072 | 0.485754 | 0.310656 |
|  |  |  | PR | **0.082782** | **0.148116** | **0.165702** | **0.003699** | **0.004931** |
|  |  | DLF | BE | 13.80017 | 12.56750 | 10.68891 | 0.487667 | 0.313123 |
|  |  |  | PR | **0.006050** | **0.011771** | **0.014995** | **0.007670** | **0.015856** |
|  | 500 | SELF | BE | 13.86485 | 12.21198 | 10.21464 | 0.492964 | 0.303773 |
|  |  |  | PR | **0.452893** | **0.782822** | **0.591641** | **0.000739** | **0.000636** |
|  |  | PLF | BE | 13.89509 | 12.20599 | 10.25567 | 0.493946 | 0.304558 |
|  |  |  | PR | **0.032562** | **0.062786** | **0.057211** | **0.001497** | **0.002073** |
|  |  | DLF | BE | 13.81803 | 12.29258 | 10.27797 | 0.493622 | 0.306700 |
|  |  |  | PR | **0.002384** | **0.005138** | **0.005482** | **0.003066** | **0.006802** |
| 30 | 50 | SELF | BE | 13.84720 | 12.66850 | 11.07980 | 0.479142 | 0.307347 |
|  |  |  | PR | **3.077080** | **4.824410** | **5.991890** | **0.005019** | **0.004279** |
|  |  | PLF | BE | 13.85340 | 13.05130 | 11.56060 | 0.482435 | 0.314924 |
|  |  |  | PR | **0.223193** | **0.381843** | **0.533656** | **0.010552** | **0.013869** |
|  |  | DLF | BE | 14.13800 | 13.14360 | 11.66740 | 0.489073 | 0.321534 |
|  |  |  | PR | **0.015905** | **0.028881** | **0.044356** | **0.021750** | **0.043761** |
|  | 100 | SELF | BE | 13.89461 | 12.49881 | 10.62590 | 0.486137 | 0.305910 |
|  |  |  | PR | **1.551918** | **2.404741** | **2.548607** | **0.002664** | **0.002265** |
|  |  | PLF | BE | 13.92163 | 12.55621 | 10.81606 | 0.489434 | 0.308882 |
|  |  |  | PR | **0.110118** | **0.187273** | **0.230604** | **0.005472** | **0.007352** |
|  |  | DLF | BE | 13.92788 | 12.65676 | 10.87924 | 0.492466 | 0.312998 |
|  |  |  | PR | **0.007898** | **0.014698** | **0.020735** | **0.011140** | **0.023621** |
|  | 200 | SELF | BE | 13.92148 | 12.26676 | 10.25838 | 0.492629 | 0.303763 |
|  |  |  | PR | **0.748405** | **1.170295** | **1.036255** | **0.001359** | **0.001156** |
|  |  | PLF | BE | 13.93816 | 12.35343 | 10.33408 | 0.493925 | 0.305617 |
|  |  |  | PR | **0.053378** | **0.093849** | **0.098372** | **0.002757** | **0.003790** |
|  |  | DLF | BE | 13.94119 | 12.43274 | 10.44372 | 0.494862 | 0.307757 |
|  |  |  | PR | **0.003918** | **0.007662** | **0.009570** | **0.005627** | **0.012416** |
|  | 500 | SELF | BE | 13.94310 | 12.13883 | 10.11571 | 0.496707 | 0.301891 |
|  |  |  | PR | **0.290880** | **0.460172** | **0.362199** | **0.000549** | **0.000466** |
|  |  | PLF | BE | 13.98279 | 12.12599 | 10.09665 | 0.497352 | 0.302438 |
|  |  |  | PR | **0.020856** | **0.037753** | **0.035176** | **0.001106** | **0.001545** |
|  |  | DLF | BE | 13.97588 | 12.16927 | 10.12850 | 0.497660 | 0.303567 |
|  |  |  | PR | **0.001498** | **0.003095** | **0.003477** | **0.002227** | **0.005092** |
