# Supplementary material for: A 3-Component Mixture of Rayleigh Distributions: Properties and Estimation in Bayesian Framework
Source: PLoS One. 2015 May 20;10(5):e0126183. doi: 10.1371/journal.pone.0126183 (PMC4439070; doi:10.1371/journal.pone.0126183)
Supplement: S6 Table — (DOCX) [file pone.0126183.s008.docx]

Table S6: The BEs and the PRs using the JP with and

|  |  | Loss Functions | | JP | | | | |
| --- | --- | --- | --- | --- | --- | --- | --- | --- |
|  |  |  |  |  |  |  |  |  |
| 25 | 50 | SELF | BE | 13.39350 | 12.67030 | 11.41530 | 0.464110 | 0.312778 |
|  |  |  | PR | **4.174710** | **6.609760** | **8.508760** | **0.006033** | **0.005202** |
|  |  | PLF | BE | 13.47350 | 12.89260 | 11.67010 | 0.470394 | 0.320900 |
|  |  |  | PR | **0.305905** | **0.509247** | **0.69107** | **0.013004** | **0.016507** |
|  |  | DLF | BE | 13.63820 | 13.11700 | 12.02360 | 0.479237 | 0.327643 |
|  |  |  | PR | **0.022167** | **0.038726** | **0.056323** | **0.027492** | **0.051343** |
|  | 100 | SELF | BE | 13.54333 | 12.44052 | 10.74275 | 0.476556 | 0.310346 |
|  |  |  | PR | **2.166230** | **3.46152** | **3.773216** | **0.003338** | **0.002869** |
|  |  | PLF | BE | 13.62400 | 12.61430 | 10.93540 | 0.481282 | 0.313582 |
|  |  |  | PR | **0.15926** | **0.274854** | **0.338587** | **0.007033** | **0.009197** |
|  |  | DLF | BE | 13.70310 | 12.70860 | 11.08050 | 0.483959 | 0.318937 |
|  |  |  | PR | **0.011750** | **0.021391** | **0.029551** | **0.014752** | **0.029040** |
|  | 200 | SELF | BE | 13.72104 | 12.31254 | 10.41913 | 0.485220 | 0.307310 |
|  |  |  | PR | **1.117253** | **1.825202** | **1.694527** | **0.001774** | **0.001522** |
|  |  | PLF | BE | 13.73624 | 12.35744 | 10.54723 | 0.486537 | 0.309606 |
|  |  |  | PR | **0.081844** | **0.145002** | **0.157900** | **0.003682** | **0.004905** |
|  |  | DLF | BE | 13.77086 | 12.54591 | 10.73238 | 0.488126 | 0.312520 |
|  |  |  | PR | **0.005989** | **0.011709** | **0.014974** | **0.007612** | **0.015808** |
|  | 500 | SELF | BE | 13.87498 | 12.15599 | 10.18852 | 0.493846 | 0.303200 |
|  |  |  | PR | **0.448022** | **0.769030** | **0.584010** | **0.000735** | **0.000631** |
|  |  | PLF | BE | 13.86561 | 12.20068 | 10.20711 | 0.493639 | 0.304991 |
|  |  |  | PR | **0.032544** | **0.062614** | **0.055742** | **0.001496** | **0.002072** |
|  |  | DLF | BE | 13.89661 | 12.20328 | 10.24223 | 0.495158 | 0.305386 |
|  |  |  | PR | **0.002324** | **0.005078** | **0.005384** | **0.003018** | **0.006768** |
| 30 | 50 | SELF | BE | 13.68670 | 12.52110 | 10.72590 | 0.479873 | 0.306706 |
|  |  |  | PR | **2.96808** | **4.58495** | **5.334450** | **0.005016** | **0.004279** |
|  |  | PLF | BE | 13.77510 | 12.73580 | 10.93250 | 0.483974 | 0.315034 |
|  |  |  | PR | **0.214693** | **0.358819** | **0.461467** | **0.010488** | **0.013831** |
|  |  | DLF | BE | 13.87720 | 12.91540 | 11.30450 | 0.488657 | 0.322428 |
|  |  |  | PR | **0.015545** | **0.027395** | **0.040838** | **0.021727** | **0.043464** |
|  | 100 | SELF | BE | 13.78005 | 12.31759 | 10.46270 | 0.487439 | 0.305450 |
|  |  |  | PR | **1.474216** | **2.252780** | **2.341053** | **0.002641** | **0.002246** |
|  |  | PLF | BE | 13.84483 | 12.49764 | 10.42876 | 0.490466 | 0.309097 |
|  |  |  | PR | **0.107058** | **0.182651** | **0.208072** | **0.005430** | **0.007339** |
|  |  | DLF | BE | 13.90681 | 12.49266 | 10.61947 | 0.493060 | 0.312634 |
|  |  |  | PR | **0.007697** | **0.014331** | **0.019433** | **0.011066** | **0.023612** |
|  | 200 | SELF | BE | 13.86261 | 12.24702 | 10.21321 | 0.492538 | 0.303907 |
|  |  |  | PR | **0.737520** | **1.149594** | **1.000934** | **0.001358** | **0.001155** |
|  |  | PLF | BE | 13.92914 | 12.24655 | 10.25669 | 0.494271 | 0.305575 |
|  |  |  | PR | **0.053137** | **0.092307** | **0.096036** | **0.002755** | **0.003785** |
|  |  | DLF | BE | 13.96508 | 12.25574 | 10.30146 | 0.496752 | 0.306433 |
|  |  |  | PR | **0.003795** | **0.007506** | **0.009232** | **0.005541** | **0.012402** |
|  | 500 | SELF | BE | 13.96350 | 12.08495 | 10.09642 | 0.497038 | 0.301567 |
|  |  |  | PR | **0.289962** | **0.454787** | **0.359298** | **0.000549** | **0.000465** |
|  |  | PLF | BE | 13.94941 | 12.15794 | 10.09697 | 0.497527 | 0.302450 |
|  |  |  | PR | **0.020826** | **0.037712** | **0.035026** | **0.001106** | **0.001545** |
|  |  | DLF | BE | 13.98841 | 12.07719 | 10.10186 | 0.498636 | 0.302595 |
|  |  |  | PR | **0.001475** | **0.003063** | **0.003441** | **0.002210** | **0.005090** |
