# Supplementary material for: A 3-Component Mixture of Rayleigh Distributions: Properties and Estimation in Bayesian Framework
Source: PLoS One. 2015 May 20;10(5):e0126183. doi: 10.1371/journal.pone.0126183 (PMC4439070; doi:10.1371/journal.pone.0126183)
Supplement: S7 Table — (DOCX) [file pone.0126183.s009.docx]

Table S7: The BEs and the PRs using the UP with and

|  |  | Loss Functions | | UP | | | | |
| --- | --- | --- | --- | --- | --- | --- | --- | --- |
|  |  |  |  |  |  |  |  |  |
| 25 | 50 | SELF | BE | 15.11570 | 15.21360 | 14.70180 | 0.446887 | 0.315727 |
|  |  |  | PR | **7.668310** | **13.42780** | **19.90600** | **0.007654** | **0.006813** |
|  |  | PLF | BE | 15.31340 | 15.61380 | 15.54320 | 0.455036 | 0.325516 |
|  |  |  | PR | **0.501003** | **0.848322** | **1.283060** | **0.017251** | **0.021270** |
|  |  | DLF | BE | 15.51450 | 15.98220 | 16.42600 | 0.461612 | 0.335228 |
|  |  |  | PR | **0.032234** | **0.053767** | **0.078823** | **0.038399** | **0.065239** |
|  | 100 | SELF | BE | 15.11800 | 14.75620 | 13.76680 | 0.460287 | 0.312265 |
|  |  |  | PR | **4.118250** | **7.027940** | **9.440140** | **0.004614** | **0.004037** |
|  |  | PLF | BE | 15.32560 | 14.88800 | 14.12520 | 0.464883 | 0.318137 |
|  |  |  | PR | **0.270099** | **0.458719** | **0.646914** | **0.010067** | **0.012600** |
|  |  | DLF | BE | 15.48550 | 15.08730 | 14.29960 | 0.472795 | 0.323096 |
|  |  |  | PR | **0.017351** | **0.030657** | **0.044418** | **0.021502** | **0.039785** |
|  | 200 | SELF | BE | 15.43531 | 14.46959 | 13.09508 | 0.473544 | 0.309107 |
|  |  |  | PR | **2.267797** | **3.794180** | **4.576478** | **0.002684** | **0.002312** |
|  |  | PLF | BE | 15.44052 | 14.62811 | 13.25725 | 0.475648 | 0.313620 |
|  |  |  | PR | **0.144151** | **0.253375** | **0.331562** | **0.005624** | **0.007216** |
|  |  | DLF | BE | 15.50953 | 14.85048 | 13.32168 | 0.479002 | 0.318504 |
|  |  |  | PR | **0.009597** | **0.017746** | **0.024852** | **0.012124** | **0.023497** |
|  | 500 | SELF | BE | 15.64398 | 14.32064 | 12.43348 | 0.485325 | 0.307325 |
|  |  |  | PR | **0.972786** | **1.704820** | **1.635986** | **0.001210** | **0.001036** |
|  |  | PLF | BE | 15.67040 | 14.31519 | 12.61697 | 0.486607 | 0.307746 |
|  |  |  | PR | **0.062393** | **0.117806** | **0.129822** | **0.002510** | **0.003332** |
|  |  | DLF | BE | 15.73962 | 14.42185 | 12.65730 | 0.488656 | 0.309417 |
|  |  |  | PR | **0.003998** | **0.008276** | **0.010127** | **0.005198** | **0.010894** |
| 30 | 50 | SELF | BE | 15.56400 | 14.80440 | 14.07450 | 0.467505 | 0.308815 |
|  |  |  | PR | **5.27490** | **8.80986** | **12.80430** | **0.005739** | **0.004918** |
|  |  | PLF | BE | 15.71080 | 15.24940 | 14.24750 | 0.474324 | 0.318034 |
|  |  |  | PR | **0.332132** | **0.572637** | **0.845632** | **0.012296** | **0.015742** |
|  |  | DLF | BE | 15.75580 | 15.43460 | 15.09480 | 0.478758 | 0.325394 |
|  |  |  | PR | **0.020762** | **0.036651** | **0.056445** | **0.025952** | **0.048931** |
|  | 100 | SELF | BE | 15.61538 | 14.58070 | 13.13655 | 0.479123 | 0.307469 |
|  |  |  | PR | **2.649712** | **4.402702** | **5.530214** | **0.003132** | **0.002671** |
|  |  | PLF | BE | 15.71521 | 14.83954 | 13.39674 | 0.480205 | 0.313373 |
|  |  |  | PR | **0.171548** | **0.296408** | **0.401634** | **0.006620** | **0.008647** |
|  |  | DLF | BE | 15.76687 | 14.88723 | 13.47946 | 0.485096 | 0.316351 |
|  |  |  | PR | **0.010754** | **0.019744** | **0.028809** | **0.013690** | **0.027485** |
|  | 200 | SELF | BE | 15.70247 | 14.31163 | 12.63754 | 0.486731 | 0.305901 |
|  |  |  | PR | **1.353830** | **2.262099** | **2.464204** | **0.001660** | **0.001406** |
|  |  | PLF | BE | 15.74906 | 14.37846 | 12.69776 | 0.488738 | 0.307630 |
|  |  |  | PR | **0.085556** | **0.154499** | **0.186951** | **0.003398** | **0.004561** |
|  |  | DLF | BE | 15.78090 | 14.49420 | 12.94743 | 0.489275 | 0.310224 |
|  |  |  | PR | **0.005491** | **0.010591** | **0.014468** | **0.007025** | **0.014771** |
|  | 500 | SELF | BE | 15.84014 | 14.16081 | 12.26866 | 0.493910 | 0.302974 |
|  |  |  | PR | **0.541392** | **0.934168** | **0.843695** | **0.000684** | **0.000581** |
|  |  | PLF | BE | 15.86928 | 14.20836 | 12.32357 | 0.494325 | 0.304086 |
|  |  |  | PR | **0.034371** | **0.065865** | **0.067514** | **0.001388** | **0.001913** |
|  |  | DLF | BE | 15.86127 | 14.29941 | 12.37318 | 0.494301 | 0.305567 |
|  |  |  | PR | **0.002198** | **0.004611** | **0.005439** | **0.002840** | **0.006308** |
