# Supplementary material for: A 3-Component Mixture of Rayleigh Distributions: Properties and Estimation in Bayesian Framework
Source: PLoS One. 2015 May 20;10(5):e0126183. doi: 10.1371/journal.pone.0126183 (PMC4439070; doi:10.1371/journal.pone.0126183)
Supplement: S8 Table — (DOCX) [file pone.0126183.s010.docx]

Table S8: The BEs and the PRs using the JP with and

|  |  | Loss Functions | | JP | | | | |
| --- | --- | --- | --- | --- | --- | --- | --- | --- |
|  |  |  |  |  |  |  |  |  |
| 25 | 50 | SELF | BE | 14.96780 | 14.57550 | 13.93930 | 0.453393 | 0.313068 |
|  |  |  | PR | **7.306700** | **12.00010** | **16.88400** | **0.007623** | **0.006667** |
|  |  | PLF | BE | 15.14660 | 15.11190 | 14.43220 | 0.459733 | 0.325297 |
|  |  |  | PR | **0.487670** | **0.814612** | **1.152940** | **0.017248** | **0.021235** |
|  |  | DLF | BE | 15.41070 | 15.50230 | 15.13280 | 0.468276 | 0.335345 |
|  |  |  | PR | **0.031401** | **0.052208** | **0.075760** | **0.037618** | **0.064932** |
|  | 100 | SELF | BE | 15.13860 | 14.48150 | 13.41110 | 0.462403 | 0.312755 |
|  |  |  | PR | **4.075790** | **6.598950** | **8.646480** | **0.004606** | **0.004010** |
|  |  | PLF | BE | 15.25440 | 14.59210 | 13.68450 | 0.470232 | 0.317048 |
|  |  |  | PR | **0.261286** | **0.440179** | **0.615404** | **0.009932** | **0.012494** |
|  |  | DLF | BE | 15.50490 | 14.84930 | 13.99560 | 0.476549 | 0.322732 |
|  |  |  | PR | **0.017062** | **0.030355** | **0.043842** | **0.021345** | **0.039716** |
|  | 200 | SELF | BE | 15.30680 | 14.46934 | 12.84924 | 0.473443 | 0.311552 |
|  |  |  | PR | **2.217586** | **3.762853** | **4.306267** | **0.002664** | **0.002309** |
|  |  | PLF | BE | 15.42961 | 14.51248 | 13.01358 | 0.477681 | 0.313408 |
|  |  |  | PR | **0.143310** | **0.251185** | **0.316505** | **0.005621** | **0.007213** |
|  |  | DLF | BE | 15.53817 | 14.65853 | 13.10938 | 0.480496 | 0.317891 |
|  |  |  | PR | **0.009418** | **0.017384** | **0.023887** | **0.011969** | **0.023147** |
|  | 500 | SELF | BE | 15.63715 | 14.26596 | 12.40257 | 0.485855 | 0.306869 |
|  |  |  | PR | **0.966013** | **1.696764** | **1.596838** | **0.001209** | **0.001036** |
|  |  | PLF | BE | 15.69649 | 14.32500 | 12.52215 | 0.487234 | 0.308702 |
|  |  |  | PR | **0.062181** | **0.117408** | **0.128202** | **0.002505** | **0.003323** |
|  |  | DLF | BE | 15.64888 | 14.41129 | 12.53421 | 0.487391 | 0.311003 |
|  |  |  | PR | **0.003992** | **0.008120** | **0.009776** | **0.005197** | **0.010760** |
| 30 | 50 | SELF | BE | 15.35800 | 14.57660 | 13.31560 | 0.471060 | 0.308452 |
|  |  |  | PR | **4.947810** | **8.266080** | **10.78650** | **0.005713** | **0.004898** |
|  |  | PLF | BE | 15.59180 | 14.89550 | 13.75830 | 0.475835 | 0.318052 |
|  |  |  | PR | **0.320650** | **0.542823** | **0.769462** | **0.012210** | **0.015686** |
|  |  | DLF | BE | 15.62690 | 15.21680 | 14.11540 | 0.480833 | 0.326659 |
|  |  |  | PR | **0.020461** | **0.035675** | **0.053454** | **0.025755** | **0.048920** |
|  | 100 | SELF | BE | 15.52212 | 14.37243 | 12.73206 | 0.481181 | 0.307124 |
|  |  |  | PR | **2.565359** | **4.211739** | **4.986836** | **0.003115** | **0.002657** |
|  |  | PLF | BE | 15.61134 | 14.54063 | 12.96967 | 0.483012 | 0.312483 |
|  |  |  | PR | **0.165003** | **0.282848** | **0.373641** | **0.006510** | **0.008535** |
|  |  | DLF | BE | 15.68469 | 14.75707 | 13.26403 | 0.485645 | 0.317044 |
|  |  |  | PR | **0.010688** | **0.019446** | **0.028039** | **0.013630** | **0.027442** |
|  | 200 | SELF | BE | 15.68846 | 14.29900 | 12.46872 | 0.487551 | 0.305483 |
|  |  |  | PR | **1.333162** | **2.232128** | **2.316557** | **0.001650** | **0.001403** |
|  |  | PLF | BE | 15.66988 | 14.32500 | 12.59988 | 0.489181 | 0.307299 |
|  |  |  | PR | **0.084804** | **0.152388** | **0.182305** | **0.003390** | **0.004560** |
|  |  | DLF | BE | 15.74674 | 14.40692 | 12.73768 | 0.490581 | 0.309792 |
|  |  |  | PR | **0.005413** | **0.010447** | **0.013925** | **0.006959** | **0.014748** |
|  | 500 | SELF | BE | 15.84955 | 14.15897 | 12.24457 | 0.493646 | 0.302983 |
|  |  |  | PR | **0.539809** | **0.927198** | **0.826315** | **0.000683** | **0.000580** |
|  |  | PLF | BE | 15.82337 | 14.25132 | 12.21530 | 0.493979 | 0.304902 |
|  |  |  | PR | **0.034370** | **0.065657** | **0.065098** | **0.001382** | **0.001911** |
|  |  | DLF | BE | 15.88106 | 14.21547 | 12.25666 | 0.495466 | 0.304863 |
|  |  |  | PR | **0.002168** | **0.004588** | **0.005290** | **0.002812** | **0.006296** |
