# Supplementary material for: A 3-Component Mixture of Rayleigh Distributions: Properties and Estimation in Bayesian Framework
Source: PLoS One. 2015 May 20;10(5):e0126183. doi: 10.1371/journal.pone.0126183 (PMC4439070; doi:10.1371/journal.pone.0126183)
Supplement: S9 Table — (DOCX) [file pone.0126183.s011.docx]

Table S9: The BEs and the PRs using the ICP with and

|  |  | Loss Functions | | ICP | | | | |
| --- | --- | --- | --- | --- | --- | --- | --- | --- |
|  |  |  |  |  |  |  |  |  |
| 25 | 50 | SELF | BE | 12.45630 | 11.53130 | 9.707880 | 0.435206 | 0.325846 |
|  |  |  | PR | **3.312110** | **5.039890** | **5.390500** | **0.004907** | **0.004415** |
|  |  | PLF | BE | 12.53190 | 11.62980 | 10.25740 | 0.439375 | 0.331690 |
|  |  |  | PR | **0.271273** | **0.424914** | **0.523750** | **0.011515** | **0.013492** |
|  |  | DLF | BE | 12.73900 | 11.74580 | 10.37850 | 0.446817 | 0.337791 |
|  |  |  | PR | **0.020888** | **0.035160** | **0.047905** | **0.025895** | **0.040265** |
|  | 100 | SELF | BE | 13.10190 | 11.87970 | 9.920830 | 0.459712 | 0.317385 |
|  |  |  | PR | **1.985420** | **3.084720** | **2.967290** | **0.003018** | **0.002636** |
|  |  | PLF | BE | 13.15130 | 11.92390 | 10.11970 | 0.463840 | 0.320522 |
|  |  |  | PR | **0.147252** | **0.245887** | **0.280877** | **0.006505** | **0.008114** |
|  |  | DLF | BE | 13.19710 | 12.11940 | 10.25300 | 0.464645 | 0.326730 |
|  |  |  | PR | **0.011589** | **0.020438** | **0.026842** | **0.014375** | **0.025195** |
|  | 200 | SELF | BE | 13.45953 | 11.94491 | 9.980061 | 0.477057 | 0.310337 |
|  |  |  | PR | **1.073586** | **1.731326** | **1.526408** | **0.001691** | **0.001455** |
|  |  | PLF | BE | 13.55520 | 11.97179 | 10.08736 | 0.478939 | 0.312791 |
|  |  |  | PR | **0.080403** | **0.142939** | **0.144576** | **0.003565** | **0.004674** |
|  |  | DLF | BE | 13.52671 | 12.09858 | 10.11109 | 0.480398 | 0.315483 |
|  |  |  | PR | **0.005954** | **0.011619** | **0.013652** | **0.007476** | **0.014842** |
|  | 500 | SELF | BE | 13.78543 | 12.04150 | 9.995411 | 0.489334 | 0.305643 |
|  |  |  | PR | **0.447490** | **0.766804** | **0.544737** | **0.000727** | **0.000628** |
|  |  | PLF | BE | 13.81800 | 11.99408 | 10.01649 | 0.490780 | 0.305941 |
|  |  |  | PR | **0.031963** | **0.061979** | **0.052350** | **0.001469** | **0.002025** |
|  |  | DLF | BE | 13.78696 | 12.08255 | 10.03669 | 0.490256 | 0.308424 |
|  |  |  | PR | **0.002311** | **0.005062** | **0.005184** | **0.003007** | **0.006634** |
| 30 | 50 | SELF | BE | 13.00440 | 11.26230 | 9.463110 | 0.448733 | 0.319055 |
|  |  |  | PR | **2.419970** | **3.285800** | **3.300220** | **0.004194** | **0.003669** |
|  |  | PLF | BE | 13.05180 | 11.62110 | 9.635240 | 0.452199 | 0.325887 |
|  |  |  | PR | **0.184446** | **0.286192** | **0.325577** | **0.009350** | **0.011429** |
|  |  | DLF | BE | 13.15230 | 11.75080 | 9.802790 | 0.456437 | 0.332063 |
|  |  |  | PR | **0.014204** | **0.024283** | **0.032602** | **0.020738** | **0.034839** |
|  | 100 | SELF | BE | 13.38790 | 11.82780 | 9.792120 | 0.468811 | 0.312783 |
|  |  |  | PR | **1.352410** | **2.007950** | **1.826850** | **0.002410** | **0.002084** |
|  |  | PLF | BE | 13.49150 | 11.76760 | 9.836090 | 0.472776 | 0.314897 |
|  |  |  | PR | **0.098723** | **0.161289** | **0.174303** | **0.005099** | **0.006598** |
|  |  | DLF | BE | 13.46030 | 11.91550 | 9.905510 | 0.474412 | 0.319080 |
|  |  |  | PR | **0.007401** | **0.013599** | **0.017114** | **0.010838** | **0.020828** |
|  | 200 | SELF | BE | 13.68132 | 11.88937 | 9.903223 | 0.483252 | 0.307062 |
|  |  |  | PR | **0.703051** | **1.067105** | **0.881765** | **0.001292** | **0.001106** |
|  |  | PLF | BE | 13.70177 | 11.90919 | 9.875911 | 0.484947 | 0.308571 |
|  |  |  | PR | **0.051009** | **0.088092** | **0.084817** | **0.002668** | **0.003588** |
|  |  | DLF | BE | 13.71986 | 11.99210 | 9.975728 | 0.486389 | 0.310386 |
|  |  |  | PR | **0.003698** | **0.007226** | **0.008501** | **0.005488** | **0.011568** |
|  | 500 | SELF | BE | 13.87626 | 11.95170 | 9.922162 | 0.493151 | 0.302904 |
|  |  |  | PR | **0.282764** | **0.440442** | **0.332440** | **0.000537** | **0.000457** |
|  |  | PLF | BE | 13.88771 | 11.99445 | 9.968509 | 0.493557 | 0.303797 |
|  |  |  | PR | **0.020424** | **0.036652** | **0.033399** | **0.001091** | **0.001508** |
|  |  | DLF | BE | 13.88609 | 11.99905 | 9.998384 | 0.494050 | 0.304388 |
|  |  |  | PR | **0.001470** | **0.003033** | **0.003336** | **0.002209** | **0.004957** |
