# Supplementary material for: A 3-Component Mixture of Rayleigh Distributions: Properties and Estimation in Bayesian Framework
Source: PLoS One. 2015 May 20;10(5):e0126183. doi: 10.1371/journal.pone.0126183 (PMC4439070; doi:10.1371/journal.pone.0126183)
Supplement: S10 Table — (DOCX) [file pone.0126183.s012.docx]

Table S10: The BEs and the PRs using the SRIGP with and

|  |  | Loss Functions | | SRIGP | | | | |
| --- | --- | --- | --- | --- | --- | --- | --- | --- |
|  |  |  |  |  |  |  |  |  |
| 25 | 50 | SELF | BE | 12.41580 | 9.872580 | 8.384830 | 0.488999 | 0.281048 |
|  |  |  | PR | **2.839060** | **3.113810** | **3.154170** | **0.003933** | **0.003148** |
|  |  | PLF | BE | 12.48690 | 10.01530 | 8.497580 | 0.492442 | 0.287191 |
|  |  |  | PR | **0.222914** | **0.292819** | **0.318114** | **0.008011** | **0.011009** |
|  |  | DLF | BE | 12.62800 | 10.09780 | 8.664570 | 0.497401 | 0.292274 |
|  |  |  | PR | **0.017388** | **0.027109** | **0.034263** | **0.016134** | **0.037716** |
|  | 100 | SELF | BE | 13.1558 | 10.79170 | 9.061650 | 0.492914 | 0.288868 |
|  |  |  | PR | **1.724270** | **2.226350** | **2.009550** | **0.002540** | **0.002076** |
|  |  | PLF | BE | 13.17320 | 10.84980 | 9.191080 | 0.495093 | 0.292048 |
|  |  |  | PR | **0.134862** | **0.205306** | **0.210979** | **0.005236** | **0.007204** |
|  |  | DLF | BE | 13.27380 | 10.92270 | 9.286450 | 0.498761 | 0.295452 |
|  |  |  | PR | **0.010312** | **0.018290** | **0.021809** | **0.010616** | **0.024481** |
|  | 200 | SELF | BE | 13.51656 | 11.36788 | 9.527406 | 0.495741 | 0.293963 |
|  |  |  | PR | **0.967750** | **1.432214** | **1.160763** | **0.001507** | **0.001255** |
|  |  | PLF | BE | 13.59065 | 11.34025 | 9.564679 | 0.498388 | 0.294961 |
|  |  |  | PR | **0.071332** | **0.121656** | **0.116161** | **0.003032** | **0.004215** |
|  |  | DLF | BE | 13.59802 | 11.45222 | 9.564555 | 0.499678 | 0.297727 |
|  |  |  | PR | **0.005360** | **0.010687** | **0.011851** | **0.006165** | **0.014293** |
|  | 500 | SELF | BE | 13.83295 | 11.68869 | 9.808748 | 0.498141 | 0.296581 |
|  |  |  | PR | **0.414568** | **0.680346** | **0.482768** | **0.000678** | **0.000572** |
|  |  | PLF | BE | 13.84977 | 11.69620 | 9.816580 | 0.499959 | 0.297356 |
|  |  |  | PR | **0.029983** | **0.057458** | **0.048030** | **0.001355** | **0.001918** |
|  |  | DLF | BE | 13.84814 | 11.74496 | 9.829517 | 0.500113 | 0.298598 |
|  |  |  | PR | **0.002166** | **0.004816** | **0.004828** | **0.002717** | **0.006399** |
| 30 | 50 | SELF | BE | 12.66920 | 10.10540 | 8.412370 | 0.489408 | 0.282957 |
|  |  |  | PR | **1.994570** | **2.192090** | **1.981290** | **0.003439** | **0.002790** |
|  |  | PLF | BE | 12.69790 | 10.30720 | 8.527430 | 0.492530 | 0.288576 |
|  |  |  | PR | **0.154614** | **0.210648** | **0.217852** | **0.007017** | **0.009788** |
|  |  | DLF | BE | 12.71220 | 10.40240 | 8.685600 | 0.496106 | 0.293461 |
|  |  |  | PR | **0.012249** | **0.019562** | **0.024478** | **0.014244** | **0.033620** |
|  | 100 | SELF | BE | 13.26541 | 10.95498 | 9.073014 | 0.493020 | 0.290529 |
|  |  |  | PR | **1.174432** | **1.497062** | **1.274877** | **0.002113** | **0.001746** |
|  |  | PLF | BE | 13.27159 | 10.99059 | 9.227898 | 0.495482 | 0.293047 |
|  |  |  | PR | **0.088369** | **0.132663** | **0.136889** | **0.004284** | **0.005981** |
|  |  | DLF | BE | 13.39458 | 11.07362 | 9.304104 | 0.497806 | 0.295777 |
|  |  |  | PR | **0.006688** | **0.011864** | **0.014290** | **0.008646** | **0.020353** |
|  | 200 | SELF | BE | 13.60888 | 11.45095 | 9.531209 | 0.496032 | 0.294721 |
|  |  |  | PR | **0.646994** | **0.911293** | **0.727342** | **0.001197** | **0.001000** |
|  |  | PLF | BE | 13.62002 | 11.52787 | 9.571352 | 0.497421 | 0.296348 |
|  |  |  | PR | **0.047605** | **0.078850** | **0.074561** | **0.002414** | **0.003387** |
|  |  | DLF | BE | 13.63246 | 11.54138 | 9.566244 | 0.498573 | 0.298283 |
|  |  |  | PR | **0.003500** | **0.006756** | **0.007593** | **0.004854** | **0.011394** |
|  | 500 | SELF | BE | 13.83580 | 11.76708 | 9.825040 | 0.498409 | 0.297611 |
|  |  |  | PR | **0.270838** | **0.407295** | **0.313079** | **0.000518** | **0.000436** |
|  |  | PLF | BE | 13.85272 | 11.79478 | 9.829630 | 0.499020 | 0.298252 |
|  |  |  | PR | **0.019707** | **0.034750** | **0.031564** | **0.001042** | **0.001468** |
|  |  | DLF | BE | 13.87027 | 11.78082 | 9.837420 | 0.499586 | 0.298968 |
|  |  |  | PR | **0.001413** | **0.002897** | **0.003174** | **0.002082** | **0.004902** |
