# Supplementary material for: A 3-Component Mixture of Rayleigh Distributions: Properties and Estimation in Bayesian Framework
Source: PLoS One. 2015 May 20;10(5):e0126183. doi: 10.1371/journal.pone.0126183 (PMC4439070; doi:10.1371/journal.pone.0126183)
Supplement: S11 Table — (DOCX) [file pone.0126183.s013.docx]

Table S11: The BEs and the PRs using the ICP with and

|  |  | Loss Functions | | ICP | | | | |
| --- | --- | --- | --- | --- | --- | --- | --- | --- |
|  |  |  |  |  |  |  |  |  |
| 25 | 50 | SELF | BE | 13.73490 | 13.29910 | 11.96900 | 0.419473 | 0.329289 |
|  |  |  | PR | **6.107610** | **9.708090** | **11.53740** | **0.006315** | **0.005847** |
|  |  | PLF | BE | 13.88590 | 13.51080 | 12.49310 | 0.428604 | 0.336993 |
|  |  |  | PR | **0.423414** | **0.672566** | **0.901702** | **0.014917** | **0.017060** |
|  |  | DLF | BE | 14.07990 | 13.78650 | 12.93430 | 0.435478 | 0.345305 |
|  |  |  | PR | **0.030383** | **0.049594** | **0.068184** | **0.035297** | **0.050900** |
|  | 100 | SELF | BE | 14.42220 | 13.73120 | 12.46710 | 0.445261 | 0.320447 |
|  |  |  | PR | **3.713760** | **6.034790** | **7.562090** | **0.004183** | **0.003721** |
|  |  | PLF | BE | 14.67580 | 14.19160 | 12.48370 | 0.451221 | 0.328136 |
|  |  |  | PR | **0.260896** | **0.439548** | **0.551260** | **0.009593** | **0.011729** |
|  |  | DLF | BE | 14.64150 | 14.28400 | 12.85210 | 0.452393 | 0.335811 |
|  |  |  | PR | **0.017013** | **0.030296** | **0.042900** | **0.021274** | **0.034830** |
|  | 200 | SELF | BE | 15.07216 | 14.08356 | 12.22602 | 0.465297 | 0.315497 |
|  |  |  | PR | **2.204722** | **3.729295** | **3.923298** | **0.002604** | **0.002286** |
|  |  | PLF | BE | 15.12101 | 14.17247 | 12.43458 | 0.467435 | 0.319082 |
|  |  |  | PR | **0.142722** | **0.250174** | **0.307937** | **0.005538** | **0.007108** |
|  |  | DLF | BE | 15.11931 | 14.25245 | 12.58989 | 0.469525 | 0.324001 |
|  |  |  | PR | **0.009253** | **0.017219** | **0.023839** | **0.011895** | **0.022106** |
|  | 500 | SELF | BE | 15.51826 | 14.06869 | 12.18885 | 0.482496 | 0.308164 |
|  |  |  | PR | **0.961318** | **1.692183** | **1.550659** | **0.001204** | **0.001035** |
|  |  | PLF | BE | 15.53602 | 14.15114 | 12.20741 | 0.483743 | 0.309943 |
|  |  |  | PR | **0.061989** | **0.115493** | **0.121111** | **0.002501** | **0.003301** |
|  |  | DLF | BE | 15.59646 | 14.20964 | 12.30022 | 0.485067 | 0.311844 |
|  |  |  | PR | **0.003861** | **0.008035** | **0.009691** | **0.005107** | **0.010610** |
| 30 | 50 | SELF | BE | 14.49390 | 13.31090 | 11.59180 | 0.439658 | 0.322682 |
|  |  |  | PR | **4.222180** | **6.308400** | **7.336960** | **0.004774** | **0.004205** |
|  |  | PLF | BE | 14.47210 | 13.60670 | 11.85160 | 0.443070 | 0.331121 |
|  |  |  | PR | **0.280918** | **0.449874** | **0.562148** | **0.010812** | **0.012860** |
|  |  | DLF | BE | 14.66200 | 13.71960 | 12.38570 | 0.450202 | 0.334728 |
|  |  |  | PR | **0.019156** | **0.032424** | **0.045212** | **0.024326** | **0.038974** |
|  | 100 | SELF | BE | 14.99840 | 13.73540 | 11.89890 | 0.462514 | 0.315004 |
|  |  |  | PR | **2.353770** | **3.732850** | **4.017950** | **0.002826** | **0.002449** |
|  |  | PLF | BE | 15.16070 | 13.91120 | 12.12480 | 0.465316 | 0.319039 |
|  |  |  | PR | **0.161711** | **0.273837** | **0.324545** | **0.006220** | **0.007841** |
|  |  | DLF | BE | 15.25060 | 13.93830 | 12.25990 | 0.468450 | 0.322999 |
|  |  |  | PR | **0.010540** | **0.018940** | **0.025566** | **0.013326** | **0.024148** |
|  | 200 | SELF | BE | 15.46960 | 13.92736 | 11.98004 | 0.478712 | 0.309097 |
|  |  |  | PR | **1.303632** | **2.137894** | **2.076094** | **0.001583** | **0.001356** |
|  |  | PLF | BE | 15.47953 | 14.07873 | 12.11888 | 0.479364 | 0.312137 |
|  |  |  | PR | **0.084060** | **0.149216** | **0.164437** | **0.003315** | **0.004351** |
|  |  | DLF | BE | 15.51559 | 14.06358 | 12.20873 | 0.481213 | 0.313922 |
|  |  |  | PR | **0.005376** | **0.010365** | **0.013272** | **0.006958** | **0.013966** |
|  | 500 | SELF | BE | 15.78673 | 13.99178 | 11.98485 | 0.490570 | 0.304575 |
|  |  |  | PR | **0.535346** | **0.919148** | **0.759252** | **0.000672** | **0.000574** |
|  |  | PLF | BE | 15.79410 | 14.06413 | 12.03446 | 0.490813 | 0.305704 |
|  |  |  | PR | **0.033856** | **0.064601** | **0.062415** | **0.001370** | **0.001874** |
|  |  | DLF | BE | 15.78968 | 14.03990 | 12.14707 | 0.492013 | 0.305867 |
|  |  |  | PR | **0.002135** | **0.004454** | **0.005186** | **0.002780** | **0.006108** |
