# Supplementary material for: A 3-Component Mixture of Rayleigh Distributions: Properties and Estimation in Bayesian Framework
Source: PLoS One. 2015 May 20;10(5):e0126183. doi: 10.1371/journal.pone.0126183 (PMC4439070; doi:10.1371/journal.pone.0126183)
Supplement: S12 Table — (DOCX) [file pone.0126183.s014.docx]

Table S12: The BEs and the PRs using the SRIGP with and

|  |  | Loss Functions | | SRIGP | | | | |
| --- | --- | --- | --- | --- | --- | --- | --- | --- |
|  |  |  |  |  |  |  |  |  |
| 25 | 50 | SELF | BE | 14.12940 | 10.76290 | 9.893980 | 0.492712 | 0.273559 |
|  |  |  | PR | **5.044590** | **5.126220** | **6.368990** | **0.004792** | **0.003657** |
|  |  | PLF | BE | 14.23720 | 10.98410 | 10.15420 | 0.496794 | 0.282475 |
|  |  |  | PR | **0.359367** | **0.433838** | **0.562368** | **0.009775** | **0.013011** |
|  |  | DLF | BE | 14.57750 | 11.10960 | 10.26720 | 0.506365 | 0.285825 |
|  |  |  | PR | **0.025465** | **0.037837** | **0.050120** | **0.019821** | **0.046209** |
|  | 100 | SELF | BE | 14.99550 | 11.93410 | 10.87760 | 0.497482 | 0.280849 |
|  |  |  | PR | **3.331750** | **3.988400** | **4.861760** | **0.003453** | **0.002639** |
|  |  | PLF | BE | 15.15810 | 12.03510 | 11.07480 | 0.503744 | 0.283401 |
|  |  |  | PR | **0.224656** | **0.322690** | **0.409807** | **0.007032** | **0.009216** |
|  |  | DLF | BE | 15.19460 | 12.36520 | 11.16100 | 0.505399 | 0.290909 |
|  |  |  | PR | **0.014872** | **0.025737** | **0.033614** | **0.014108** | **0.032071** |
|  | 200 | SELF | BE | 15.47070 | 12.85042 | 11.33553 | 0.499634 | 0.288866 |
|  |  |  | PR | **1.969310** | **2.835682** | **2.932961** | **0.002237** | **0.001772** |
|  |  | PLF | BE | 15.46878 | 12.97985 | 11.54214 | 0.502849 | 0.292578 |
|  |  |  | PR | **0.129176** | **0.213224** | **0.248005** | **0.004547** | **0.006010** |
|  |  | DLF | BE | 15.60051 | 13.02921 | 11.68024 | 0.504586 | 0.293552 |
|  |  |  | PR | **0.008427** | **0.016342** | **0.020485** | **0.009183** | **0.020589** |
|  | 500 | SELF | BE | 15.79459 | 13.48256 | 11.74429 | 0.499973 | 0.294583 |
|  |  |  | PR | **0.885659** | **1.480003** | **1.300546** | **0.001086** | **0.000895** |
|  |  | PLF | BE | 15.82909 | 13.50333 | 11.78925 | 0.501719 | 0.295470 |
|  |  |  | PR | **0.056355** | **0.107154** | **0.106849** | **0.002181** | **0.002980** |
|  |  | DLF | BE | 15.83188 | 13.59719 | 11.95292 | 0.501406 | 0.297713 |
|  |  |  | PR | **0.003617** | **0.007850** | **0.008980** | **0.004428** | **0.010002** |
| 30 | 50 | SELF | BE | 14.27580 | 11.34740 | 10.17060 | 0.492754 | 0.278738 |
|  |  |  | PR | **3.372410** | **3.749020** | **4.345180** | **0.003781** | **0.002999** |
|  |  | PLF | BE | 14.41020 | 11.70620 | 10.21220 | 0.493553 | 0.287044 |
|  |  |  | PR | **0.238820** | **0.314828** | **0.373074** | **0.007755** | **0.010674** |
|  |  | DLF | BE | 14.60880 | 11.78840 | 10.40410 | 0.498823 | 0.290531 |
|  |  |  | PR | **0.016454** | **0.025819** | **0.033339** | **0.015626** | **0.037193** |
|  | 100 | SELF | BE | 15.02220 | 12.53500 | 11.02280 | 0.493137 | 0.288115 |
|  |  |  | PR | **2.098020** | **2.781770** | **2.889410** | **0.002431** | **0.001978** |
|  |  | PLF | BE | 15.16400 | 12.60550 | 11.09150 | 0.496542 | 0.291268 |
|  |  |  | PR | **0.138829** | **0.210161** | **0.243476** | **0.004922** | **0.006769** |
|  |  | DLF | BE | 15.22070 | 12.73230 | 11.06240 | 0.499154 | 0.294432 |
|  |  |  | PR | **0.009183** | **0.016368** | **0.020307** | **0.009901** | **0.023230** |
|  | 200 | SELF | BE | 15.55212 | 13.19190 | 11.38182 | 0.496154 | 0.294060 |
|  |  |  | PR | **1.176212** | **1.742179** | **1.597941** | **0.001418** | **0.001175** |
|  |  | PLF | BE | 15.51102 | 13.32577 | 11.47114 | 0.497446 | 0.296102 |
|  |  |  | PR | **0.076264** | **0.129405** | **0.137065** | **0.002877** | **0.003981** |
|  |  | DLF | BE | 15.61242 | 13.39281 | 11.51781 | 0.499153 | 0.298270 |
|  |  |  | PR | **0.004946** | **0.009686** | **0.011393** | **0.005800** | **0.013456** |
|  | 500 | SELF | BE | 15.83318 | 13.65357 | 11.75611 | 0.498662 | 0.297036 |
|  |  |  | PR | **0.500638** | **0.824685** | **0.679479** | **0.000631** | **0.000531** |
|  |  | PLF | BE | 15.86186 | 13.69871 | 11.75073 | 0.499537 | 0.297910 |
|  |  |  | PR | **0.031816** | **0.059915** | **0.056366** | **0.001270** | **0.001786** |
|  |  | DLF | BE | 15.85072 | 13.72905 | 11.86169 | 0.499945 | 0.298604 |
|  |  |  | PR | **0.002028** | **0.004376** | **0.004842** | **0.002560** | **0.005999** |
