# Supplementary material for: A 3-Component Mixture of Rayleigh Distributions: Properties and Estimation in Bayesian Framework
Source: PLoS One. 2015 May 20;10(5):e0126183. doi: 10.1371/journal.pone.0126183 (PMC4439070; doi:10.1371/journal.pone.0126183)
Supplement: S13 Table — (DOCX) [file pone.0126183.s015.docx]

**Table S13:** The BEs and the PRs using the UP, the JP, the ICP and the SRIGP under SELF, PLF and DLF.

| Prior | Loss Function | |  |  |  |  |  |
| --- | --- | --- | --- | --- | --- | --- | --- |
| UP | SELF | BE | 13.283002 | 12.976803 | 17.076093 | 0.668545 | 0.259355 |
|  |  | PR | **0.071691** | **0.196332** | **2.236985** | **0.000182** | **0.000151** |
|  | PLF | BE | 13.285700 | 12.984366 | 17.141469 | 0.668681 | 0.259647 |
|  |  | PR | **0.005397** | **0.015125** | **0.130751** | **0.000272** | **0.000583** |
|  | DLF | BE | 13.288399 | 12.991933 | 17.207094 | 0.668817 | 0.259939 |
|  |  | PR | **0.000406** | **0.001164** | **0.007613** | **0.000406** | **0.002244** |
| JP | SELF | BE | 13.285888 | 12.968821 | 16.964059 | 0.668737 | 0.259374 |
|  |  | PR | **0.071567** | **0.196040** | **2.197074** | **0.000181** | **0.000151** |
|  | PLF | BE | 13.288581 | 12.976377 | 17.028693 | 0.668872 | 0.259665 |
|  |  | PR | **0.005386** | **0.015112** | **0.129267** | **0.000271** | **0.000583** |
|  | DLF | BE | 13.291274 | 12.983937 | 17.093573 | 0.669008 | 0.259957 |
|  |  | PR | **0.000405** | **0.001164** | **0.007577** | **0.000406** | **0.002244** |
| ICP | SELF | BE | 13.285347 | 12.867944 | 16.114958 | 0.667142 | 0.260323 |
|  |  | PR | **0.070390** | **0.191481** | **1.899696** | **0.000180** | **0.000150** |
|  | PLF | BE | 13.287996 | 12.875382 | 16.173792 | 0.667277 | 0.260612 |
|  |  | PR | **0.005298** | **0.014876** | **0.117669** | **0.000270** | **0.000578** |
|  | DLF | BE | 13.290646 | 12.882824 | 16.232842 | 0.667412 | 0.260901 |
|  |  | PR | **0.000399** | **0.001155** | **0.007262** | **0.000404** | **0.002217** |
| SRIGP | SELF | BE | 13.278964 | 12.851172 | 16.215158 | 0.666797 | 0.258982 |
|  |  | PR | **0.070748** | **0.189718** | **1.955075** | **0.000179** | **0.000148** |
|  | PLF | BE | 13.281628 | 12.858551 | 16.275332 | 0.666931 | 0.259269 |
|  |  | PR | **0.005327** | **0.014758** | **0.120348** | **0.000268** | **0.000573** |
|  | DLF | BE | 13.284292 | 12.865934 | 16.335729 | 0.667065 | 0.259556 |
|  |  | PR | **0.000401** | **0.001147** | **0.007381** | **0.000402** | **0.002208** |
